# Host relationships and geographic distribution of species of *Acanthobothrium* Blanchard, 1848 (Onchoproteocephalidea, Onchobothriidae) in elasmobranchs: a metadata analysis

**DOI:** 10.3897/zookeys.940.46352

**Published:** 2020-06-11

**Authors:** Francisco Zaragoza-Tapia, Griselda Pulido-Flores, Scott L. Gardner, Scott Monks

**Affiliations:** 1 Universidad Autónoma del Estado de Hidalgo, Centro de Investigaciones Biológicas, Apartado Postal 1-10, C.P. 42001, Pachuca, Hidalgo, México Universidad Autónoma del Estado de Hidalgo Hidalgo Mexico; 2 Harold W. Manter Laboratory of Parasitology, University of Nebraska-Lincoln, Lincoln, NE 68588-0514, USA University of Nebraska Lincoln United States of America

**Keywords:** Biodiversity, Elasmobranchii, Eucestoda, geographic distribution, rays, sharks

## Abstract

Species of *Acanthobothrium* have been documented as parasites of the spiral intestine of elasmobranchs. Results of a metadata analysis indicate that 114 species of elasmobranchs have been reported as hosts of 200 species of *Acanthobothrium*. The metadata analysis revealed that 3.7% of species of sharks and 14.9% of species of rays that have been reported as hosts to date; some species are parasitized by more than one species of *Acanthobothrium*. This work provides a Category designation, as proposed by Ghoshroy and Caira (2001), for each species of *Acanthobothrium*. These Category designations are a tool to facilitate comparisons among members of *Acanthobothrium* for descriptions of new species in the future.

## Introduction

According to Last et al. (2016b), there are 34 families comprised of 516 valid species of sharks and 26 families that include 633 valid species of rays. Since that publication, six new species of sharks and rays were described by: Yokota and Carvalho (2017) (two species of rays), Vaz and Carvalho (2018) (one species of shark), Rutledge (2019) (one species of ray), Grace et al. (2019) (one species of shark) and Concha et al. (2019) (one species of ray). This brought the current number of recognized species to 517 species of sharks and 637 species of rays.

Elasmobranchs (sharks, skates and rays) are host to a great variety of parasites in nature, particularly helminths. *Acanthobothrium* Blanchard, 1848 (Onchoproteocephalidea) is the most diverse genus that has been reported as parasite of the spiral intestine of elasmobranchs (Caira and Jensen 2017). At the present time, 201 species of *Acanthobothrium* are considered to be valid (Maleki et al. 2013; Caira and Jensen 2017; Rodríguez-Ibarra et al. 2018; Franzese and Ivanov 2018; Maleki et al. 2019; Zaragoza-Tapia et al. 2019, 2020). The genus consists of species that exclusively parasitize elasmobranchs as adults and, in many cases, individual species are thought to parasitize only a single species of elasmobranch (Caira 2011; Caira and Jensen 2017). Therefore, the genus *Acanthobothrium* is an excellent model for future studies of host-parasite co-speciation.

The main goal of this work is to provide a revised checklist based on a metadata analysis of the host relationships of members of *Acanthobothrium* and their geographic distribution based on records that have been generated from different parts of the world. The checklist focuses on the 201 valid species of *Acanthobothrium* and reports correlated with the genera and species of elasmobranchs, and includes the geographical distribution of each.

**Figure 1.**
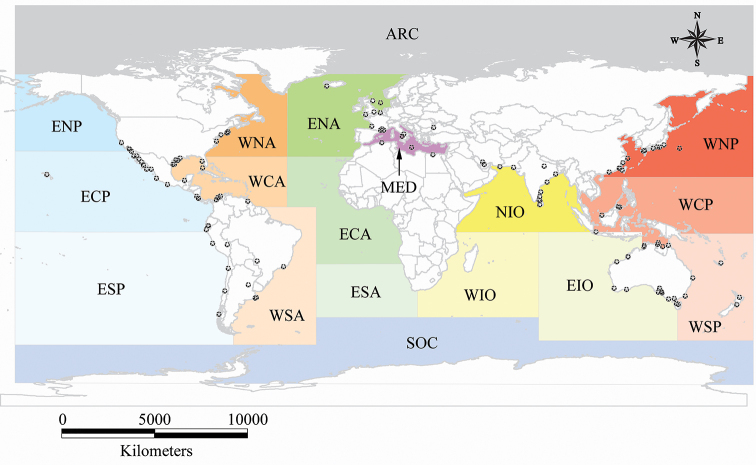
Type localities of species of *Acanthobothrium* reported worldwide and the biogeographic regions (Last et al. 2016b) of the geographic distribution of their hosts (see Table 1).

The number of species of *Acanthobothrium* continues to grow and there are still regions of the world without a single report of this genus (see Figure 1). For some time, the process of distinguishing new species of *Acanthobothrium* from existing species has become more and more unwieldy because of the large number of species. As an identification tool, Ghoshroy and Caira (2001) developed a categorical method for identifying species for initial comparisons. Therefore, in order to provide an update to this method, categorical designations are provided in the present checklist for each species of *Acanthobothrium* in the manner proposed by Ghoshroy and Caira (2001). The categories are based on and obtained from the combination of four quantitative characters: total length of the worm; the number of proglottids comprising the strobila; the number of testes per proglottid; and symmetry of the ovarian lobes. This categorical designation allows parasitologists working with this genus to postulate a group of similar species, those of the same category designation, for comparison of a new species or to aid in the preliminary identification of known species. As an additional aid, in the checklist the accession number, if known, of type specimens of each species is provided.

## Materials and methods

The checklist, updated until March 2020, was based on bibliographical information from two sources of information: 1. a compilation of the records of species of *Acanthobothrium* as originally described, complemented by information gathered from Global Cestode Database (Caira et al. 2019) and from recent compilation studies (e.g., Ghoshroy and Caira 2001; Campbell and Beveridge 2002; Fyler and Caira 2006; Caira and Jensen 2017); and 2. information for the distribution and taxonomy of elasmobranchs that integrated a bibliographical search using different databases of literature published to date (e.g., Del Moral-Flores et al. 2015; Last et al. 2016b; Merlo-Serna and García-Prieto 2016; Alves et al. 2017) and data from FishBase (Froese and Pauly 2019).

In the checklist, the species of *Acanthobothrium* are arranged in alphabetical order. The scientific names and geographic distribution of elasmobranchs have been updated based on Last et al. (2016a, 2016b), Amaral et al. (2018) and Froese and Pauly (2019). The regional classification scheme of the geographic distribution of the hosts is according to Last et al. (2016b) with additional information from Froese and Pauly (2019). The following abbreviations are used for biogeographic regions (see Figure 1):

**ARC** Arctic Ocean;

**ECA** Eastern Central Atlantic;

**ECP** Eastern Central Pacific;

**EIO** Eastern Indian Ocean;

**ENA** Eastern North Atlantic;

**ENP** Eastern North Pacific;

**ESA** Eastern South Atlantic;

**ESP** Eastern South Pacific;

**MED** Mediterranean Sea;

**NIO** Northern Indian Ocean;

**SOC** Southern (Antarctic) Ocean;

**WCA** Western Central Atlantic;

**WCP** Western Central Pacific;

**WIO** Western Indian Ocean;

**WNA** Western North Atlantic;

**WNP** Western North Pacific;

**WSA** Western South Atlantic;

**WSP** Western South Pacific.

Information for each species of *Acanthobothrium* presented herein includes the name of the species, authority (original description referenced in the literature cited), abbreviation of the name of the collection where specimens are deposited and the accession numbers of the specimens, followed by the status of the specimens (holotype, paratype, neotype, syntype or voucher). The acronym “NR” was used for data that are not reported in the original source. Localities (type or/and additional localities) were given and referenced in the literature cited. A Category designation was supplied for all species using the categorical method proposed by Ghoshroy and Caira (2001).

The categorical method was developed as a system of grouping species of *Acanthobothrium* based on the combination of four qualitative characters: the total length of worms- ≤ 15 mm = S (short) or > 15 mm = L (long); the number of proglottids comprising the strobila- ≤ 50 = F (few) or > 50 = M (many); the number of testes per proglottid- ≤ 80 = F (few) or > 80 = M (many); and symmetry of the ovarian lobes- symmetrical = S or asymmetrical = A. Of the possible combinations the following 10 categories currently are recognized and coded as follows: 1 = SFFS; 2 = SFFA; 3 = LMMA; 4 = LMMS; 5 = LMFS; 6 = LMFA; 7 = LFFA; 8 = SMFS; 9 = LFFS; 10 = SMMS. This method limited the number of necessary comparisons required in the description between known species with new species assigned to the same Category. For this work, the categories and characteristics were used as in Ghoshroy and Caira (2001) and Fyler and Caira (2006) but the character values are as given in the original descriptions or as supplemented by the most recent taxonomic publications. In the Category designation, the type species is identified by number for this classification; the symbol “–” was used for the additional reports of species with additional hosts and/or localities.

For specimens deposited in a formal collection, acronyms are as follows:

**AMS**Australian Museum, Sydney;

**CH-MHNJP** Colecciones Helmintológicas del Museo de Historia Natural “Javier Prado” y del Instituto de Medicina Tropical “Daniel. A. Carrión”, Universidad Mayor de San Marcos, Perú;

**CHE** Colección de Helmintos, Centro de Investigaciones Biológicas, Universidad Autónoma del Estado de Hidalgo, Pachuca, México;

**CHIOC**Coleção Helmintológica do Instituto Oswaldo Cruz, Rio de Janeiro, Brazil;

**CNHE** Colección Nacional de Helmintos del Instituto de Biología, Universidad Nacional Autónoma de México, México;

**DMNZ** Dominion Musem (=National Museum), New Zealand;

**DZAUW** Department of Zoology, Andhra University, Waltair, India;

**DZCJ** Department of Zoology, Bipin Bihari, P. G. College, Jhansi, India;

**HWML**University of Nebraska State Museum, Harold W. Manter Laboratory, Division of Parasitology, Lincoln, Nebraska, United States;

**IPCAS** Institute of Parasitology, Academy of Sciences of the Czech Republic, České Budějovice, Czech Republic;

**IPMB** Institut Penyelidikan Marin Borneo (Borneo Marine Research Institute), Universiti Malaysia Sabah, Kota Kinabalu, Sabah, Malaysia;

**LRP** Lawrence R. Penner Parasitology Collection, Helminthological Collection, University of Connecticut, Storrs, Connecticut, United States;

**MACN-Pa**Museo Argentino de Ciencias Naturales, Colección Parasitológica, Buenos Aires, Argentina;

**MEPN** Museum of the Escuela Politecnica Nacional, Quito, Ecuador;

**MHNLS**Museo de Historia Natural La Salle, Caracas, Venezuela;

**MHNP** Museo de Historia Natural, Lima, Peru;

**MLP** Museo de Ciencias Naturales de La Plata, Departamento de Zoología Invertebrados (Parasitología), Argentina;

**MNHG** Museum of Natural History, Geneva, Switzerland;

**MNHN** Muséum National d’Histoire Naturelle, Paris;

**MNHNC**Museo Nacional de Historia Natural de Chile;

**MPM** Meguro Parasitology Museum, Tokyo, Japan;

**MZUM (P)** Muzium Zoologi, Universiti Malaya, Kuala Lumpur, Malaysia;

**MZUSP**Museu de Zoologia da Universidade de São Paulo, Brazil;

**NHMUK**The Natural History Museum, London;

**NMNS** National Museum of Natural Science, Taichung, Taiwan;

**PRLXU**Parasitology Research Laboratory, Xiamen University, China;

**QM**Queensland Museum, Brisbane, Queensland, Australia;

**SAM AHC**South Australian Museum, Adelaide, Australia;

**SBC** Sarawak Biodiversity Center, Kuching, Sarawak, Malaysia;

**SPUK**School of Parasitology, Department of Zoology, University of Karachi, Pakistan;

**SYSU** School of Life Sciences, Sun Yat-sen University;

**UAA** Department of Zoology, University of Allahabad, Allahabad, India;

**USNPC**United States National Parasite Collection, Beltsville, Maryland, United States;

**ZCUOK** Zoological Collection, University of Kurdistan, Sanandaj, Iran;

**ZIMC** Collection of the Zoological Survey of India, Indian Museum, Calcutta and the Collection of the Department of Zoology, the University of Allahabad, India;

**ZMB** Natural History Museum Berlin, Germany;

**ZUTC**Collection of the Zoological Museum, University of Tehran, Tehran, Iran.

## Results

The information obtained from the metadata analysis (Table 1) is comprised of 336 reports of the 201 valid species of *Acanthobothrium*. The list includes the type host of each species, type locality, and additional hosts and/or localities. Five of the elasmobranchs that were reported as hosts of *Acanthobothrium* were only identified to genus and four others are reported as “cf.” (= similar to) (see Table 1).

**Table 1.** Species of *Acanthobothrium* reported from the different species of elasmobranchs of the world. Abbreviations: Gd = Geographical distribution; Ht = Holotype; Nt = Neotype; Pt = Paratype; Va = Voucher; Loc = Locality; Sou = Source; Cd = Category designation; * = Additional host; † = Additional locality; ‡ = Category designation obtained from Ghoshroy and Caira (2001); § = Category designation obtained from Fyler and Caira (2006); ¶ = Category designation obtained in this study from original descriptions; ** = Host identification requiring confirmation.

Species of *Acanthobothrium*	Ht	Nt, Pt or Va	Species of Host	Gd	Loc	Sou	Cd
*A. adlardi* Campbell & Beveridge, 2002	SAM AHC 28210	SAM AHC 22723, 22724	*Pristiophorus cirratus* (Latham, 1794)	EIO, WSP	Port Stanvac, South Australia	Campbell and Beveridge (2002)	4§
*A. aetiobatidis* (Shipley, 1900) Southwell, 1925	NR	NR	Aetobatus narinari** (Euphrasen, 1790)	WSA, WCA, WNA, ECA	Lifu, Loyalty Islands	Shipley (1900),Southwell (1925), Baer and Euzet (1962),Goldstein (1967)	6§
*A. amazonensis* Mayes, Brooks & Thorson, 1978	USNPC 74806	USNPC 74807; HWML 20562	*Potamotrygon circularis* German, 1913	WSA	Itacuari River, Brazil	Mayes et al. (1978)	5‡
*A. americanum* Campbell, 1969	USNPC 71355	USNPC 71356	*Hypanus americanus* (Hildebrand & Schroeder, 1928)	WSA, WCA, WNA	Chesapeake Bay, Virginia, USA	Campbell (1969)	6‡
*A. americanum*†	NR	NR	*Hypanus americanus*	WSA, WCA, WNA	Isla Margarita, Venezuela	Mayes and Brooks (1981)	–
*A. angelae* Campbell & Beveridge, 2002	SAM AHC 22661	SAM AHC 22709, 22712	*Hypnos monopterygius* (Shaw, 1795)	EIO, WSP	Yarraville Shoals, South Australia	Campbell and Beveridge (2002)	5§
*A. annapinkiensis* Carvajal & Goldstein, 1971	MNHNC 20.003	NR	*Zearaja chilensis* (Guichenot, 1848)	ESP, WSA,	Anna Pink Bay, Chile	Carvajal-G. and Goldstein (1971)	2‡
*A. arlenae* Campbell & Beveridge, 2002	SAM AHC 28225	SAM AHC 28226	Aetobatus narinari**	WSA, WCA, WNA, ECA	Fog Bay, Timor Sea, North Australia	Campbell and Beveridge (2002)	6§
*A. asnihae* Fyler & Caira, 2006	MZUM (P) 142	USNPC 96413; LRP 3809-3812, LRP 3814 (including cross sections and SEM specimens); MZUM (P) 143–144; IPMB 77.14.04	*Urogymnus polylepis* (Bleeker, 1852)	NIO, WCP	Off Kampung Abai, Kinabatangan River, Sabah, Malaysia	Fyler and Caira (2006)	1§
*A. asrinae* Maleki, Malek & Palm, 2015	ZUTC 1325	ZUTC 1326; ZMB E.7569; SEM voucher ZUTC 1327	Rhynchobatus cf. djiddensis** (Forsskå, 1775)	WIO, NIO	Persian Gulf, Iran	Maleki et al. (2015)	**1**¶
*A. atahualpai* Marques, Brooks & Barringa, 1997	MEPN 3029	MNHG 22098; CNHE 3029	*Gymnura afuerae* (Hildebrand, 1946)	ECP, ESP	Puerto Bolivar, Provincia de El Oro, Ecuador	Marques et al. (1997a)	1‡
*A. australis* Robinson, 1965	AMS	AMS	*Squalus megalops* (Macleay, 1881)	ENA, MED, ECA, ESA, WIO, EIO, WSP	Eden, New South Wales, Australia	Robinson (1965)	3§
*A. australis*†	NR	SAM AHC 22696	*Squalus megalops*	ENA, MED, ECA, ESA, WIO, EIO, WSP	Beachport, South Australia	Campbell and Beveridge (2002)	–
*A. bajaensis* Appy & Dailey, 1973	USNPC 72567	USNPC 72568	*Heterodontus francisci* (Girard, 1855)	ECP, ESP	San Quintin Bay, Baja California, Mexico	Appy and Dailey (1973)	4‡
*A. bajaensis*†	NR	NR	*Heterodontus francisci*	ECP, ESP	Newport Bay, California, USA	Appy and Dailey (1973)	–
*A. bartonae* Campbell & Beveridge, 2002	SAM AHC 28235	NR	Rhynchobatus djiddensis**	WIO, NIO	Broome, Western Australia	Campbell and Beveridge (2002)	1§
*A. batailloni* Euzet, 1955	NR	NR	*Myliobatis aquila* (Linnaeus, 1758)	ENA, MED, ECA, ESA, WIO	Mediterranean Sea, Gulfe du Lion	Euzet (1955)	7(2)‡
A. batailloni*†	NR	MNHNC 20015	Myliobatis chilensis** Philippi, 1892	ESP	Antofagasta, Chile	Carvajal-G. and Jeges-G. (1980)	–
A. batailloni*†	NR	NR	Myliobatis chilensis**	ESP	Coquimbo, Chile	Carvajal-G. and Jeges-G. (1980)	–
A. batailloni*†	NR	NR	Myliobatis chilensis**	ESP	Trujillo, Peru	Escalante-A. (1986)	–
*A. benedenii* (Lönnberg, 1889)	NR	NR	*Raja clavata* Linnaeus, 1758	ENA, MED, ECA, ESA, WIO	Mediterranean Sea	Lönnberg (1889)	**2**¶
A. benedenii*†	NR	NR	Pteroplatytrygon violacea** (Bonaparte, 1832)	ENP, ECP, ESP, WSA, WCA, WNA, ENA, MED, ECA, ESA, WIO, NIO, EIO, WSP, WCP, WNP	Naples, Italy	Baer (1948)	–
A. benedenii*†	NR	NR	Torpedo marmorata** Risso, 1810	ENA, MED, ECA, ESA	Casablanca, Marruecos	Euzet (1952), Euzet (1959)	–
*A. bengalense* Baer & Euzet, 1962	NR	NR	*Pastinachus sephen* (Forsskål, 1775)	NIO	Nagapattinam, India	Baer and Euzet (1962)	4§
*A. blairi* Campbell & Beveridge, 2002	SAM AHC 28211	SAM AHC 28212	*Dipturus whitleyi* (Iredale, 1938)	EIO, WSP	Stanley, Tasmania	Campbell and Beveridge (2002)	3§
*A. blairi*†	NR	NR	*Dipturus whitleyi*	EIO, WSP	Spencer Gulf, South Australia	Campbell and Beveridge (2002)	–
*A. bobconniorum* Fyler & Caira, 2010	QM G232499	QM G232500–G232501; USNPC 104278; LRP 7583–7585; cross sections of one paratype worm and voucher LRP 7586, 7588, 7589, SEM LRP 7587–7590	Rhynchobatus laevis** (Bloch & Schneider, 1801)	NIO, WNP	Gove Harbor, Gulf of Carpentaria, Northern Territory, Australia	Fyler and Caira (2010)	**2**¶
*A. brachyacanthum* Riser, 1955	USNPC 37418	NR	*Raja stellulata* (Gilbert, 1915)	ENP, ECP	Monterey Bay, California, USA	Riser (1955)	2‡
A. brachyacanthum*	NR	NR	Beringraja binoculata** (Gilbert, 1855)	ENP, ECP	Monterey Bay, California, USA	Riser (1955)	–
*A. brayi* Campbell & Beveridge, 2002	SAM AHC 22670	SAM AHC 22730	*Sutorectus tentaculatus* (Peters, 1864)	EIO, WSP	Eastern Shoal, South Australia	Campbell and Beveridge (2002)	2§
*A. brevissime* Linton, 1909	USNPC 9008	NR	*Hypanus say* (Lesueur, 1817)	WSA, WCA, WNA	Dry Tortugas, Florida, USA	Linton (1908), Goldstein (1964)	2‡
A. brevissime*†	NR	NR	*Raja eglanteria* Bosc, 1800	WCA, WNA	Gulf of Mexico, Chesapeake Bay, Virginia, USA	Campbell (1969)	–
A. brevissime*†	NR	USNPC 71349, 71350	*Hypanus americanus*	WSA, WCA, WNA	Gulf of Mexico, Chesapeake Bay, Virginia, USA	Campbell (1969)	–
A. brevissime*†	NR	CH-MHNJP 727	Myliobatis peruvianus** Garman, 1913	ESP	Lima, Peru	Tantaleán-Vidaurre (1991)	–
*A. brevissime*†	USNPC 9008	USNPC 60178 (neotype)	*Hypanus say*	WSA, WCA, WNA	Gulf of Mexico, Chesapeake Bay, Virginia, USA	Campbell (1969), Vardo-Zalik and Campbell (2011)	–
*A. bullardi* Ghoshroy & Caira, 2001	CNHE 4045	CNHE 4046–4047; LRP 2060–2062; USNPC 90466–90468	*Hypanus dipterurus* (Jordan & Gilbert, 1880)	ECP	Bahía de Los Angeles, Gulf of California, Mexico	Ghoshroy and Caira (2001)	2‡
*A. bullardi*†	NR	NR	*Hypanus dipterurus*	ECP	Puertecitos, Gulf of California, Mexico	Ghoshroy and Caira (2001)	–
*A. bullardi*†	NR	NR	*Hypanus dipterurus*	ECP	Santa Rosalia, Gulf of California, Mexico	Ghoshroy and Caira (2001)	–
*A. cairae* Vardo-Zalik & Campbell, 2011	USNPC 103801	USNPC 103802–103814	*Bathytoshia centroura* (Mitchill, 1815)	WSA, WCA, WNA	Narragansett Bay off Sakonnet Point, Rhode Island, USA	Vardo-Zalik and Campbell (2011)	**3**¶
*A. campbelli* Marques, Brooks & Monks, 1995	MNHG 20014	MNHG 20015–20016; HWML 38546; CNHE 3033	*Urotrygon chilensis* (Günther, 1872)	ECP, ESP	Costa de Pajaros, Puntarenas, Costa Rica	Marques et al. (1995)	2‡
A. campbelli*†	NR	MEPN 3033	*Hypanus longus* (Garman, 1880)	ECP	Puerto Huatulco, Provincia de El Oro, Ecuador	Marques et al. (1997a)	–
*A. cannoni* Campbell & Beveridge, 2002	SAM AHC 28236	SAM AHC 28237	*Himantura uarnak* (Gmelin, 1789)	WIO, NIO, EIO, WCP	Fog Bay, Timor Sea, North Australia	Campbell and Beveridge (2002)	4§
*A. cartagenensis* Brooks & Mayes, 1980	USNPC 75159	NR	*Urobatis jamaicensis* (Cuvier, 1816)	WCA, WNA	Cartagena, Colombia	Brooks and Mayes (1980)	**1**¶
*A. cartagenensis*†	NR	CNHE 9706; HWML 101020; CHE P00061	*Urobatis jamaicensis*	WCA, WNA	Ría Lagartos, Yucatán, Quintana Roo	Monks et al. (2015)	–
*A. cartagenensis*†	NR	CNHE 9706; HWML 101020; CHE P00061	*Urobatis jamaicensis*	WCA, WNA	Isla Contoy, Quintana Roo	Monks et al. (2015)	–
*A. cartagenensis*†	NR	CNHE 9706; HWML 101020; CHE P00061	*Urobatis jamaicensis*	WCA, WNA	Isla Cozumel, El Paso de los Cedros, Quintana Roo	Monks et al. (2015)	–
*A. cartagenensis*†	NR	CNHE 9706; HWML 101020; CHE P00061	*Urobatis jamaicensis*	WCA, WNA	Xcalak, Quintana Roo	Monks et al. (2015)	–
*A. cestraciontis* (Yamaguti, 1934)	NR	NR	*Heterodontus japonicus* Miklouho-Maclay & Macleay, 1884	WNP, WCP	Pacific Ocean, Japan	Yamaguti (1934)	4§
*A. cestraciontis*†	NR	NR	Sphyraena japonica** (Bloch &Schneider, 1801)	?	Pacific Ocean, Japan	Goldstein (1967)	–
*A. chabahariense* Maleki, Malek & Rastgoo, 2018	ZCUOK 100	ZCUOK 101– 112 and (SME specimen) ZCUOK 113	Pastinachus cf. sephen**	NIO	Chabahar coasts, the coast of the Gulf of Oman, Iran	Maleki et al. (2018)	**1**¶
*A. chengi* Cornford, 1974	USNPC 72958	USNPC 72959	*Bathytoshia lata* (Garman, 1880)	ECP, ENA, MED, ECA, WIO, NIO, EIO, WSP, WCP, WNP	Oahu, Hawaii	Cornford (1974)	3§
*A. chilensis* Rego, Vincente & Herrera, 1968	CHIOC 30.308 a-c	NR	Sarda chiliensis** (Cuvier, 1832)	?	Paita, Piúra, Peru	Rêgo et al. (1968)	3‡
*A. chisholmae* Campbell & Beveridge, 2002	SAM AHC 28223	SAM AHC 28224	Pastinachus sephen**	NIO	Nickol Bay, Western Australia	Campbell and Beveridge (2002)	2§
*A. cimari* Marques, Brooks & Monks, 1995	MNHG 20017	MNHG 20018–20020; HWML 38547	*Hypanus longus*	ECP	Punta Morales, Puntarenas Province, Costa Rica	Marques et al. (1995)	2‡
*A. clarkeae* Campbell & Beveridge, 2002	SAM AHC 28349	SAM AHC 28350	*Urolophus paucimaculatus* Dixon, 1969	EIO, WSP	Queenscliff, Victoria, Australia	Campbell and Beveridge (2002)	1§
A. clarkeae*†	NR	SAM AHC 28243, 28244	*Urolophus cruciatus* (Lacepède, 1804)	EIO, WSP	Devonport, Tasmania	Campbell and Beveridge (2002)	–
A. clarkeae*†	NR	SAM AHC 28208	*Urolophus expansus* McCulloch, 1916	EIO	Beachport, South Australia	Campbell and Beveridge (2002)	–
*A. cleofanus* Monks, Brooks & Lonce de Leon, 1996	CNHE 2670	CNHE 2671; MNHG 38576; HWML 38576.	*Hypanus longus*	ECP	Chamela Bay, Jalisco, Mexico	(Monks et al. 1996)	3‡
*A. colombianum* Brooks & Mayes, 1980	USNPC 75160	USNPC 75161	*Aetobatus narinari*	WSA, WCA, WNA, ECA	Cartagena, Colombia	Brooks and Mayes (1980)	9‡
*A. confusum* Baer & Euzet, 1962	NR	NR	Neotrygon kuhlii** (Müller & Henle, 1841)	WSP	Indian Ocean, Sri Lanka	Baer and Euzet (1962)	5§
*A. coquimbensis* Carvajal & Jeges, 1980	MNHNC 20016	NR	*Myliobatis chilensis*	ESP	Antofagasta, Chile	Carvajal-G. and Jeges-G. (1980)	2‡
*A. coquimbensis*†	NR	NR	*Myliobatis chilensis*	ESP	Coquimbo, Chile	Carvajal-G. and Jeges-G. (1980)	–
*A. coronatum* (Rudolphi, 1819), Blanchard, 1848	NR	NR	*Dipturus batis* (Linnaeus, 1758)	ENA	Mediterranean Sea, Italy	Rudolphi (1819), Baer (1948)	4§
A. coronatum*	NR	NR	*Scyliorhinus stellaris* (Linnaeus, 1758)	ENA, MED, ECA	Mediterranean Sea, Italy	Rudolphi (1819), Baer (1948)	–
A. coronatum*	NR	NR	*Torpedo marmorata*	ENA, MED, ECA, ESA	Mediterranean Sea, Italy	Rudolphi (1819), Baer (1948)	–
A. coronatum*	NR	NR	*Torpedo torpedo* (Linnaeus, 1758)	ENA, MED, ECA	Mediterranean Sea, Italy	Rudolphi (1819), Baer (1948)	–
A. coronatum*	NR	NR	*Dasyatis pastinaca* (Linnaeus, 1758)	ENA, MED, ECA	Mediterranean Sea, Italy	Rudolphi (1819), Baer (1948)	–
A. coronatum*†	NR	NR	Hemitrygon akajei** (Müller & Henle, 1841)	WNP	Nakatsu, West Japan	Yoshida (1917)	–
A. coronatum*†	NR	NR	Aetobatus narinari**	WSA, WCA, WNA, ECA	Batavia, Java, Indonesia	MacCallum (1921)	–
A. coronatum*†	NR	NR	*Scyliorhinus stellaris*	ENA, MED, ECA	Sète, France	Euzet (1959)	–
A. coronatum*†	NR	NR	*Scyliorhinus stellaris*	ENA, MED, ECA	Concarneau, France	Euzet (1959)	–
A. coronatum*†	NR	NR	*Mustelus mustelus* (Linnaeus, 1758)	ENA, MED, ECA, ESA	Naples, Italy	Euzet (1959)	–
A. coronatum*†	NR	NR	*Scyliorhinus stellaris*	ENA, MED, ECA	Cardigan Bay, UK	Rees and Williams (1965)	–
A. coronatum*	NR	NR	*Carcharodon carcharias* (Linnaeus, 1758)	MED	Mediterranean Sea	Goldstein (1967)	–
A. coronatum*†	NR	MNHG 40003, 40009	*Scyliorhinus canicula* (Linnaeus, 1758)	ENA, MED, ECA	Naples, Italy	Euzet (1959), Vardo-Zalik and Campbell (2011)	–
*A. costarricense* Marques, Brooks & Monks, 1995	MNHG 20008	MNHG 20009–20010; HWML 38544; CNHE 3034	*Hypanus longus*	ECP	Punta Morales, Puntarenas Province, Costa Rica	Marques et al. (1995)	2‡
*A. costarricense*†	NR	MEPN 3034	*Hypanus longus*	ECP	Puerto Huatulco, Provincia de El Oro, Ecuador	Marques et al. (1997a)	–
*A. crassicolle* Wedl, 1855	NR	MNHG 40014 88/77	*Dasyatis pastinaca*	ENA, MED, ECA	Arcacho, Gironde, France	Dollfus (1926), Baer (1948), Goldstein (1967)	3§
*A. cribbi* Campbell & Beveridge, 2002	SAM AHC 28251	SAM AHC 28252	*Gymnura australis* (Ramsay & Ogilby, 1886)	EIO, WSP, WCP	Gulf of Carpentaria, Northern Territory, Australia	Campbell and Beveridge (2002)	4§
*A. dasi* Ghoshroy & Caira, 2001	CNHE 4043	CNHE 4044; HWML 15549–15551; LRP 2051–2054; USNPC 90463–90465	*Hypanus dipterurus*	ECP	Puertecitos, Gulf of California, Mexico	Ghoshroy and Caira (2001)	2‡
*A. dasybati* Yamaguti, 1934	NR	NR	*Hemitrygon akajei*	WNP	Tarumi, Kobe, Japan	Yamaguti (1934)	4§
A. dasybati*†	NR	NR	Okamejei kenojei** (Müller & Henle, 1841)	WNP	Maisaka, Japan	Yamaguti (1952)	–
A. dasybati*†	NR	NR	*Urolophus* sp.** (*U. fuscus*?)	?	Hamazima, Mie, Japan	Yamaguti (1952)	–
*A. dighaensis* Srivastava & Capoor, 1980	UAA	NR	*Pateobatis uarnacoides* (Bleeker, 1852)	NIO, WCP	Digha, Orissa, India	Srivastav and Capoor (1980)	4§
*A. dollyae* Caira & Burge, 2001	CNHE 4169	CNHE 4170; LRP 2097–2101; USNPC 90837–90839	*Diplobatis ommata* (Jordan and Gilbert, 1890)	ECP	Bahía de Los Angeles, Gulf of California, Mexico	Caira and Burge (2001)	**1**¶
*A. dollyae*†	NR	NR	*Diplobatis ommata*	ECP	Isla San Esteban, Gulf of California, Mexico	Caira and Burge (2001)	–
*A. dollyae*†	NR	NR	*Diplobatis ommata*	ECP	Punta Arena, Gulf of California, Mexico	Caira and Burge (2001)	–
*A. dujardini* van Beneden, 1850	NR	NR	*Raja clavata*	ENA, MED, ECA, ESA, WIO	English Channel,Belgium	van Beneden (1850), Goldstein (1967)	2§
*A. dujardini*	NR	NR	*Raja clavata*	ENA, MED, ECA, ESA, WIO	English Channel, Belgium	Williams (1969)	–
A. dujardini*†	NR	NR	Raja brachyura** Lafont, 1871	ENA, MED, ECA	Roscoff, France	Euzet (1959)	–
A. dujardini*†	NR	NR	Raja montagui** Fowler, 1910	ENA, MED	British Isles	Williams (1960)	–
*A. dysbiotos* (MacCallum, 1921) Williams, 1969	NR	NR	Aetobatus narinari**	WSA, WCA, WNA, ECA	Batavia, Java, Indonesia	MacCallum (1921), Williams (1969)	4§
*A. edmondsi* Campbell & Beveridge, 2002	SAM AHC 28205	SAM AHC 28206, 22704	*Parascyllium ferrugineum* McCulloch, 1911	EIO, WSP	Port Stanvac, South Australia	Campbell and Beveridge (2002)	5§
*A. edmondsi*†	NR	NR	*Parascyllium ferrugineum*	EIO, WSP	Holdfast Bay, South Australia	Campbell and Beveridge (2002)	–
*A. edmondsi*†	NR	NR	*Parascyllium ferrugineum*	EIO, WSP	Esperance, Western Australia	Campbell and Beveridge (2002)	–
*A. edwardsi* Williams, 1969	NR	NR	*Leucoraja fullonica* (Linnaeus, 1758)	ENA, MED, ARC	West coast of Britain, United Kingdom	Williams (1969)	2§
*A. electricolum* Brooks & Mayes, 1978	USNPC 74728	USNPC 74729	*Narcine brasiliensis* (Olfers, 1831)	WSA	Caribbean Sea, near Cartagena, Colombia	Brooks and Mayes (1978)	9‡
*A. elongatum* Subhapradha, 1955	NR	NR	*Rhynchobatus djiddensis*	WIO, NIO	Madras Coast, India	Subhapradha (1955)	**4**¶
*A. etini* Fyler & Caira, 2006	MZUM (P) 145	USNPC 96414–96415; LRP 3815-3824 (including cross sections and SEM specimens); MZUM (P) 146; IPMB 77.14.05	*Urogymnus polylepis*	NIO, WCP	Off Kampung Abai, Kinabatangan River, Sabah, Malaysia	Fyler and Caira (2006)	8§
*A. filicolle* (Zschokke, 1888) Yamaguti, 1959	NR	NR	*Torpedo marmorata*	ENA, MED, ECA, ESA	Mediterranean Sea	Zschokke (1888), Yamaguti (1959b)	**1(8)**¶
A. filicolle*	NR	NR	*Torpedo torpedo*	ENA, MED, ECA	Mediterranean Sea	Williams (1969)	–
*A. floridensis* Goldstein, 1964	USNPC 60025	NR	*Raja eglanteria*	WCA, WNA	Gulf of Mexico and Coast of Massachusetts	Goldstein (1964)	8(10)‡
A. floridensis*†	NR	USNPC 103848–103850	*Raja texana* Chandler, 1921	WCA	Gulf of Mexico	Vardo-Zalik and Campbell (2011)	–
*A. floridensis*†	NR	NR	*Raja eglanteria*	WCA, WNA	Gulf of Mexico, Chesapeake Bay, Virginia, USA, USA	Campbell (1969)	–
*A. fogeli* Goldstein, 1964	USNPC 60024	NR	*Gymnura micrura* (Bloch & Schneider, 1801)	WSA, WCA, WNA, ECA	Northeastern Gulf of Mexico, Florida	Goldstein (1964)	1‡
*A. fogeli*†	NR	NR	*Gymnura micrura*	WSA, WCA, WNA, ECA	Isla Margarita, Venezuela	Mayes and Brooks (1981)	–
*A. foulki* Reyda & Caira, 2006	MZUM (P) 168(h)	USNPC 97463–97464; LRP 3850–3853 (including cross sections and SEM specimens); MZUM (P) 169(p)–171(p); IPMB 77.08.14	*Pateobatis uarnacoides*	NIO, WCP	Off Kampung Tetabuan, Sabah, Malaysia	Reyda and Caira (2006)	**1**¶
*A. franus* Marques, Centritto & Stewart, 1997	CNHE 3139	USNPC 87374; CHIOC 33754a, b; CNHE 3140	*Narcine entemedor* Jordan & Starks, 1895	ECP	Cuajiniquil Beach, Gulf of Santa Helena, Guanacaste, Costa Rica	Marques et al. (1997b)	5(8)‡
*A. fylerae* Maleki, Malek & Palm, 2015	ZUTC 1319	ZUTC 1320–1323; ZMB E.7568; SEM voucher ZUTC 1324	Rhynchobatus cf. djiddensis**	WIO, NIO	Gulf of Oman, Iran	Maleki et al. (2015)	**1**¶
*A. gasseri* Campbell & Beveridge, 2002	SAM AHC 28217	SAM AHC 28218	Pastinachus sephen**	NIO	Nickol Bay, Western Australia	Campbell and Beveridge (2002)	3§
*A. gibsoni* Campbell & Beveridge, 2002	SAM AHC 28239	NR	Rhynchobatus djiddensis**	WIO, NIO	Fog Bay, Timor Sea, North Australia	Campbell and Beveridge (2002)	3§
*A. giganticum* Sanaka, Lakshmi & Hanumantharao, 1993	NR	NR	Gymnura micrura**	WSA, WCA, WNA, ECA	Waltair coast, India	Sanaka et al. (1993)	5§
*A. gloveri* Campbell & Beveridge, 2002	SAM AHC 22600	SAM AHC 22715	*Trygonorrhina fasciata* Müller & Henle, 1841	WSP	Goolwa, South Australia	Campbell and Beveridge (2002)	2§
*A. gnomus* Reyda & Caira, 2006	MZUM (P) 172(h)	USNPC 97465–97466; LRP 3854–3859 (includes cross sections and SEM specimens); MZUM (P) 173(p)–175(p); IPMB 77.08.15	*Pateobatis uarnacoides*	NIO, WCP	Off Kampung Tetabuan, Sabah, Malaysia	Reyda and Caira (2006)	**1**¶
*A. goldsteini* Appy & Dailey, 1973	USNPC 72569	USNPC 72570	*Platyrhinoidis triseriata* (Jordan & Gilbert, 1880)	ENP, ECP	Seal Beach, California, USA	Appy and Dailey (1973)	5(9)‡
*A. gonzalesmugaburoi* Severino & Sarmiento, 1979	CH-MHNJP 340	CH-MHNJP 341, 341a, 341b	*Myliobatis peruvianus*	ESP	Callao, Lima, Peru	Severino and Sarmiento (1979)	**7(6)**¶
*A. gracile* Yamaguti, 1952	NR	NR	*Narke japonica* (Temminck & Schlegel, 1850)	WNP	Tokushima, Japan	Yamaguti (1952)	3§
*A. grandiceps* Yamaguti, 1952	MPM 22638	NR	*Telatrygon zugei* (Müller & Henle, 1841)	WCP, WNP	East China Sea, Japan	Yamaguti (1952), Yang et al. (2016)	4§
A. grandiceps*	NR	NR	*Hemitrygon akajei*	WNP	East China Sea, Japan	Yamaguti (1952)	–
*A. guanghaiense* Yang, Sun, Zhi, Iwaki, Reyda & Yang, 2016	MPM 21229	MPM 21230; SYSU 20140818-1-4	*Hemitrygon akajei*	WNP	Off Guanghai Port, Taishan, Guangdong Province, China	Yang et al. (2016)	**2**¶
*A. halehae* Maleki, Malek & Palm, 2019	ZCUOK 127	ZCUOK 128–130; ZUTC Platy. 1342–1343, 1 SEM voucher ZUTC Platy. 1344	Gymnura cf. poecilura** (Shaw, 1804)	NIO, EIO, WCP, WNP	Chabahar coast, Gulf of Oman, Iran	Maleki et al. (2019)	**1**¶
*A. hanumantharaoi* Rao, 1977	NR	NR	*Aetomylaeus nichofii* (Bloch & Schneider, 1801)	NIO, EIO, WCP, WNP	Waltair coast, Benegal Bay, India	Rao (1977)	4§
*A. herdmani* Southwell, 1912	NR	NR	Neotrygon kuhlii**	WSP	Ceylon Pearl Bank, Sri Lanka	Southwell (1912), Southwell (1925), Southwell (1930)	**3**¶
*A. heterodonti* Drummond, 1937	NR	NR	*Heterodontus portusjacksoni* (Meyer, 1793)	EIO, WSP	Lady Julia Perey Island, Victoria, Australia	Drummond (1937)	4§
*A. heterodonti*†	NR	SAM AHC 22595, 22597, 15744	*Heterodontus portusjacksoni*	EIO, WSP	Derwent Estuary, Hobart, Tasmania	Campbell and Beveridge (2002)	–
*A. heterodonti*†	NR	NR	*Heterodontus portusjacksoni*	EIO, WSP	Bunbury, Western Australia	Campbell and Beveridge (2002)	–
*A. himanturi* Brooks, 1977	USNPC 73963	USNPC 73964; HWML 20260	*Styracura schmardae* (Werner, 1904)	WCA	Caribbean Sea, La Cienaga, Magdalena, Colombia	Brooks (1977)	1‡
*A. hispidum* Riser, 1955	USNPC 37416	NR	*Tetronarce californica* (Ayres, 1855)	ENP, ECP, WNP	Monterey Bay, California, USA	Riser (1955)	5‡
*A. holorhini* Alexander, 1953	USNPC 47853	USNPC 47854	*Myliobatis californicus* Grill, 1865	ENP, ECP	Long Beach Harbor, California, USA	Alexander (1953)	3‡
A. holorhini*†	NR	CHIMTDC 542	*Myliobatis chilensis*	ESP	Callao, Peru	Rodriguez and Tantaleán-Vidaurre (1980)	–
*A. hypanus* Zaragoza-Tapia, Pulido-Flores & Monks, 2020	CNHE 11255	CNHE 11256; HWML 216261	*Hypanus longus*	ECP	La Puntilla, Mazatlán, Sinaloa, Mexico	Zaragoza-Tapia et al. (2020)	**2**¶
*A. hypermekkolpos* Fyler & Caira, 2010	QM G232506	QM G232507; USNPC 104280; LRP 7591, hologenophores LRP 7592–7593	Rhynchobatus laevis**	NIO, WNP	Gove Harbor, Gulf of Carpentaria, Northern Territory, Australia	Fyler and Caira (2010)	**1**¶
*A. icelandicum* Manger, 1972	NR	NR	*Dipturus batis*	ENA	Faxa Bay, Western coasts Iceland	Manger (1972)	3§
*A. ijimai* Yoshida, 1917	NR	MPM 22639	*Hemitrygon akajei*	WNP	Tokyo, Japan	Yoshida (1917), Williams (1969),Yang et al. (2016)	4§
*A. ijimai*†	NR	NR	*Hemitrygon akajei*	WNP	East China Sea, Japan	Yamaguti (1952)	–
*A. inbiorium* Marques, Centritto & Stewart, 1997	CNHE 3137	USNPC 87373; CHIOC 33753a, b; CNHE 3138	*Narcine entemedor*	ECP	Cuajiniquil Beach, Gulf of Santa Helena, Guanacaste, Costa Rica	Marques et al. (1997b)	5‡
*A. incognita* (MacCallum, 1921) Wardle & McLeod, 1952	NR	NR	*Dasyatis pastinaca*	ENA, MED, ECA	New York Aquarium	MacCallum (1921), Southwell (1925), Williams (1969)	?¶
*A. indicum* (Subhapradha, 1955)	NR	NR	Narcine brasiliensis**	WSA	Madras Coast, India	Subhapradha (1955), Williams (1969)	5§
*A. intermedium* Perrenoud, 1931	NR	NR	Dasyatis pastinaca**	ENA, MED, ECA	Tauranga, New Zealand	Perrenoud (1931)	4§
*A. jalalii* Maleki, Malek & Palm, 2013	ZUTC 1291	ZUTC 1292–1295), SEM voucher ZUTC 1296); IPCAS C–639); ZMB E.7559	Pastinachus cf. sephen**	NIO	Gulf of Oman, Iran	Maleki et al. (2013)	**1**¶
*A. jamesi* Maleki, Malek & Palm, 2015	ZUTC 1328	ZMB E.7570; SEM voucher ZUTC 1329.	Rhynchobatus cf. djiddensis**	WIO, NIO	Persian Gulf, Iran	Maleki et al. (2015)	**1**¶
*A. janineae* Maleki, Malek & Palm, 2015	ZUTC 1311	ZUTC 1312–1316; ZMB E.7566; SEM vouchers ZUTC 1317–1318	Rhynchobatus cf. djiddensis**	WIO, NIO	Gulf of Oman, Iran	Maleki et al. (2015)	**1**¶
*A. jeanneae* Fyler & Caira, 2010	QM G232502	QM G232503–G232505; USNPC 104279; LRP 7573–7575, cross sections of one paratype worm and voucher LRP 7580–7582, SEM LRP 7576–7578, hologenophore LRP 7579	Rhynchobatus laevis**	NIO, WNP	Gove Harbor, Gulf of Carpentaria, Northern Territory, Australia	Fyler and Caira (2010)	**1**¶
*A. jonesi* Campbell & Beveridge, 2002	SAM AHC 28227	SAM AHC 28228	*Dasyatis* sp.**	?	Cape Ford, North Australia	Campbell and Beveridge (2002)	6§
*A. karachiense* Bilqees, 1980	NR	SPUK 2000 (syntype)	*Mustelus manazo* Bleeker, 1855	NIO, WCP, WNP	Karachi Coast, Pakistan	Bilqees (1980)	4§
*A. kurdistanense* Maleki, Malek & Palm, 2019	ZCUOK 122	ZCUOK 123–127; ZUTC Platy. 1336–1340, 1 SEM voucher ZUTC Platy. 1341	Gymnura cf. poecilura**	NIO, EIO, WCP, WNP	Chabahar coast, Gulf of Oman, Iran	Maleki et al. (2019)	**1**¶
*A. larsoni* Reyda & Caira, 2006	MZUM (P) 176(h)	USNPC 97467–97468; LRP 3860–3865 (including cross sections and SEM specimens); MZUM (P) 177(p)–180(p); IPMB 77.08.16	*Pateobatis uarnacoides*	NIO, WCP	Off Kampung Tetabuan, Sabah, Malaysia	Reyda and Caira (2006)	**1**¶
*A. lasti* Campbell & Beveridge, 2002	SAM AHC 28247	SAM AHC 28248	Rhynchobatus djiddensis**	WIO, NIO	Broome, Western Australia	Campbell and Beveridge (2002)	2§
*A. latum* Yamaguti, 1952	MPM 22637	NR	*Hemitrygon akajei*	WNP	Sea of Ariake, Kyusyu, Japan	Yamaguti (1952), Yang et al. (2016)	4§
*A. laurenbrownae* Campbell & Beveridge, 2002	SAM AHC 28215	SAM AHC 28216	*Pastinachus sephen*	NIO	Nickol Bay, Western Australia	Campbell and Beveridge (2002)	1§
*A. lentiginosum* Vardo-Zalik & Campbell, 2011	USNPC 103815	USNPC 103816–103819	*Pseudobatos lentiginosus* (Garman, 1880)	WCA, WNA	Gulf of Mexico	Vardo-Zalik and Campbell (2011)	**1**¶
*A. lepidum* Reyda & Caira, 2006	MZUM (P) 181(h)	USNPC 97469; LRP 3866–3868 (including cross sections and SEM specimens); MZUM (P) 182(p)–183(p); IPMB 77.08.17	*Pateobatis uarnacoides*	NIO, WCP	Off Kampung Tetabuan, Sabah, Malaysia	Reyda and Caira (2006)	**1**¶
*A. lilium* Baer & Euzet, 1962	NR	NR	*Dasyatis* sp.**	?	Ceylon Pearl Bank, Sri Lanka	Baer and Euzet (1962)	2§
*A. lineatum* Campbell, 1969	USNPC 71353	USNPC 71354	*Hypanus americanus*	WSA, WCA, WNA	Chesapeake Bay, Virginia, USA	Campbell (1969)	1‡
*A. lintoni* Goldstein, Henson & Schlicht, 1968	USNPC 62938	USNPC 62939	Narcine brasiliensis**	WSA	Gulf of Mexico, Texas, USA	Goldstein et al. (1969)	1(8,9,5)‡
*A. lintoni*†	NR	USNPC 74851	Narcine brasiliensis**	WSA	Gulf of Mexico, Florida, USA	Goldstein et al. (1969)	–
*A. longipedunculata* Meheswari, Sanaka, Lakshmi & Rao, 1985	NR	NR	*Himantura uarnak*	WIO, NIO, EIO, WCP	Waltair coast, India	Maheswari et al. (1985)	6§
*A. lusarmientoi* Severino & Verano, 1980	CH-MHNJP 342	CH-MHNJP 343, 343a	*Sympterygia brevicaudata* (Cope, 1877)	ECP, ESP	Callao, Lima, Peru	Severino and Verano (1980)	**7**¶
*A. macracanthum* Southwell, 1925	NR	NR	*Urogymnus* sp.**	?	Madras Coast, India	Southwell (1925)	6§
*A. macrocephalum* Wang & Yang, 2001	MPM 21231	NR	*Hemitrygon akajei*	WNP	Xiamen, Fujiari, China	Wang and Yang (2001), Yang et al. (2016)	4§
*A. macrocephalum*†	MPM 21231	MPM 21232; SYSU 20140620-1-7	*Hemitrygon akajei*	WNP	Off Guanghai Port, Guangdong, China	Yang et al. (2016)	–
*A. macrocephalum*†	NR	NR	*Hemitrygon akajei*	WNP	Sanya Fishing Port, Sanya, Hainan, China	Yang et al. (2016)	–
*A. maculatum* Riser, 1955	USNPC 37417	NR	*Myliobatis californicus*	ENP, ECP	Monterey Bay, California, USA	Riser (1955)	6(3)‡
*A. magnum* Euzet, 1959	NR	NR	*Pteroplatytrygon violacea*	ENP, ECP, ESP, WSA, WCA, WNA, ENA, MED, ECA, ESA, WIO, NIO, EIO, WSP, WCP, WNP	Mediterranean Sea, France	Euzet (1959)	4§
*A. makranense* Maleki, Malek & Palm, 2019	ZCUOK 130	ZCUOK 131–135; ZUTC Platy. 1345–1350, 1 SEM voucher ZCUOK 139, 1 SEM voucher ZUTC Platy. 1350	Gymnura cf. poecilura**	NIO, EIO, WCP, WNP	Chabahar coast, Gulf of Oman, Iran	Maleki et al. (2019)	**1**¶
*A. manteri* Hassan, 1983	IHAHE S1051/A	IHAHE S1051/B	Pastinachus sephen**	NIO	Mediterranean Sea, Egypt	Hassan (1983)	5§
*A. margieae* Fyler, 2011	NMNS 6356–001	NMNS 6356–002, 6356–003, 6356–004, 6356–005, 6356–006, 6356–007; LRP 7468–7477; USNPC 103274	*Orectolobus japonicus* Regan, 1906	WNP, WCP	Off Penghu Island, East China Sea, Magong, Taiwan	Fyler (2011)	**8**¶
*A. marplatensis* Ivanov & Campbell, 1998	MLP 4025	MLP 4026; USNMPC 87475; NHMUK 1998.2.10.1-2	*Atlantoraja castelnaui* (Miranda Ribeiro, 1907)	WSA	Mar del Plata, Buenos Aires, Argentina	Ivanov and Campbell (1998)	1‡
*A. marquesi* Rodríguez-Ibarra, Pulido-Flores, Violante-González & Monks, 2018	CNHE 10554	CNHE 10555, 10556; HWML 139377–139384; CHE P00061–P00063	Aetobatus cf. narinari**	WSA, WCA, WNA, ECA	Laguna de Términos, Ciudad del Carmen, Campeche, Mexico	Rodríguez-Ibarra et al. (2018)	**3**¶
*A. marquesi*†	NR	NR	Aetobatus cf. narinari**	WSA, WCA, WNA, ECA	Champotón, Campeche, Mexico	Rodríguez-Ibarra et al. (2018)	–
*A. martini* Campbell & Beveridge, 2002	SAM AHC 28213	SAM AHC 28214	*Myliobatis tenuicaudatus* Hector, 1877	EIO, WSP	Bunbury, Western Australia	Campbell and Beveridge (2002)	1§
*A. maryanskii* Caira & Burge, 2001	CNHE 4171	CNHE 4172; LRP 2012, 2013; USNPC 90840, 90841	*Diplobatis ommata*	ECP	Loreto, Gulfo of California, Mexico	Caira and Burge (2001)	**5**¶
*A. marymichaelorum* Twohig, Caira & Fyler, 2008	MZUM(P) 699(H)	MZUM(P) 700(P)–702(P); SBC P–00028; USNPC 100700; LRP 4162–4164 (whole mount), 4167–4168 (cross sections)	*Brevitrygon walga* (Müller & Henle, 1841)	NIO	Off Sematan, Sarawak, Malaysia	Twohig et al. (2008)	**1**¶
*A. marymichaelorum*	NR	NR	*Brevitrygon walga*	NIO	Off Mukah, Sarawak, Malaysia.	Twohig et al. (2008)	–
*A. masnihae* Fyler & Caira, 2006	MZUM (P) 147	USNPC 96416–96417; LRP 3825-3835 (including cross sections and SEM specimens); MZUM (P) 148; IPMB 77.14.06	*Urogymnus polylepis*	NIO, WCP	Kampung Abai, Kinabatangan River, Sabah, Malaysia	Fyler and Caira (2006)	2§
*A. mathiasi* Euzet, 1959	NR	NR	*Mustelus mustelus*	ENA, MED, ECA, ESA	Sète, France	Euzet (1959)	1§
A. mathiasi*	NR	NR	*Mustelus canis* (Mitchill, 1815)	WNA, WCA, WSA	Sète, France	Euzet (1959)	–
*A. matttaylori* Fyler & Caira, 2010	QM G232508	Hologenophore USNPC 104281	Rhynchobatus laevis**	NIO, WNP	Gove Harbor, Gulf of Carpentaria, Northern Territory, Australia	Fyler and Caira (2010)	**4**¶
*A. micracantha* Yamaguti, 1952	NR	MPM 22635, 22636	*Hemitrygon akajei*	WNP	Nagasaki, East China Sea, Japan	Yamaguti (1952), Yang et al. (2016)	4§
A. micracantha*	NR	NR	Gymnura micrura**	WSA, WCA, WNA, ECA	Nagasaki, East China Sea, Japan	Yamaguti (1952)	–
A. micracantha*	NR	NR	*Telatrygon zugei*	WCP, WNP	Nagasaki, East China Sea, Japan	Yamaguti (1952)	–
*A. microcephalum* Alexander, 1953	USNPC 47852	NR	*Myliobatis californicus*	ENP, ECP	Long Beach Harbor, California, USA	Alexander (1953)	4‡
*A. minus* Tazerouti, Kechemir-Issad & Euzet, 2009	MNHN HEL 76, Th 180	MNHN HEL 77, Th 181, HEL 78, Th 182, HEL 79, Th 183; NHMUK 2009.2.10.1-2	*Raja asterias* Delaroche, 1809	ENA, MED	Cap Djinet, Algérie	Tazerouti et al. (2009)	**2**¶
*A. minus*†	NR	NR	*Raja asterias*	ENA, MED	Zemmouri El Bahri, Algérie	Tazerouti et al. (2009)	–
*A. minus*†	NR	NR	*Raja asterias*	ENA, MED	Bouharoun, Algérie	Tazerouti et al. (2009)	–
*A. minusculus* Marques, Brooks & Barriga, 1997	MEPN 3030	MNHG 22099; HWML 39178, CNHE 3030	*Urobatis tumbesensis* (Chirichigno F. & McEachran, 1979)	ECP	Puerto Hualtaco, Provincia de El Oro, Ecuador	Marques et al. (1997a)	1‡
*A. monksi* Marques, Brooks & Barriga, 1997	MEPN 3031	MNHG 22100; HWML 39179; CNHE 3031	Aetobatus narinari**	WSA, WCA, WNA, ECA	Puerto Jelí, Provincia de El Oro, Ecuador	Marques et al. (1997a)	1‡
*A. mooreae* Campbell & Beveridge, 2002	SAM AHC 28209	SAM AHC 22665, 22718, 28265	*Trygonorrhina fasciata*	WSP	Northhaven, South Australia	Campbell and Beveridge (2002)	2§
*A. mujibi* Bilqees, 1980	NR	SPUK 2001 (syntype)	*Mustelus manazo*	NIO, WCP, WNP	Karachi Coast, Pakistan	Bilqees (1980)	?¶
*A. musculosum* (Baer, 1948) Yamaguti, 1959	NR	NR	*Pteroplatytrygon violacea*	ENP, ECP, ESP, WSA, WCA, WNA, ENA, MED, ECA, ESA, WIO, NIO, EIO, WSP, WCP, WNP	New Zealand	Baer (1948), Euzet (1959), Yamaguti (1959a),Williams (1969)	4§
*A. myliomaculata* Srivastav, Shweta & Noopur, 1995	DZCJ	NR	*Aetomylaeus maculatus* (Gray, 1834)	NIO, WCP, WNP	Madras Coast, India	Srivastav et al. (1995)	4§
*A. nanogravidum* Zschoche, Caira & Fyler, 2011	QM G232166	QM G232167–G23217, cross sections QM G232171, G23217; USNPC 104103); LRP 7480–7483, cross sections LRP 7486–7491, SEM LRP 7484–7485), egg mounts LRP 7492–7493	*Pastinachus ater* (Macleay, 1883)	WIO, NIO, EIO, WSP, WCP	Gulf of Carpentaria off Weipa, Queensland, Australia.	Zschoche et al. (2011)	**1**¶
*A. nicoyaense* Brooks & McCorquodale, 1995	USNPC 84477	USNPC 84388; MNHG 18255	Aetobatus narinari**	WSA, WCA, WNA, ECA	Punta Morales, Golfo de Nicoya, Costa Rica	Brooks and McCorquodale (1995)	1‡
*A. ningdense* Yang, Sun, Zhi, Iwaki, Reyda & Yang, 2016	MPM 21226	MPM 21227, 21228; SYSU 20121113-1-3, 20141002-1-27	*Hemitrygon akajei*	WNP	Fuhai aquatic market, Ningde, Fujian Province, China	Yang et al. (2016)	**4**¶
*A. ningdense*†	NR	NR	*Hemitrygon akajei*	WNP	Off Wanjichi aquatic wholesale market, Taizhou, Zhejiang Province, China	Yang et al. (2016)	–
*A. ningdense*†	NR	NR	*Hemitrygon akajei*	WNP	8^th^ Seafood Market, Xiamen, Fujian Province, China	Yang et al. (2016)	–
*A. ningdense*†	NR	NR	*Hemitrygon akajei*	WNP	Guanghai Port, Taishan, Guangdong Province, China	Yang et al. (2016)	–
*A. ningdense*†	NR	NR	*Hemitrygon akajei*	WNP	Sanya Fishing Port, Sanya, Hainan Province, China	Yang et al. (2016)	–
*A. obuncus* Marques, Brooks & Barriga, 1997	MEPN 3032	MNHG 22101; HWML 39180; CNHE 3032, 3167	*Hypanus longus*	ECP	Puerto Hualtaco, Provincia de El Oro, Ecuador	Marques et al. (1997a)	6‡
*A. ocallaghani* Campbell & Beveridge, 2002	SAM AHC 28202	SAM AHC 28203	*Aptychotrema vincentiana* (Haacke, 1885	EIO	Musgrave Shoal, South Australia	Campbell and Beveridge (2002)	2§
*A. oceanharvestae* Fyler, Caira & Jensen, 2009	QM 231345	QM G231346–G231347; USNPC 101957–101958; LRP 4317–4318; cross sections QM 231349, QM G231348; SEM LRP 4319–4320, 4327–4328, hologenophores LRP 4321, LRP 4322	*Urogymnus acanthobothrium* Last, White & Kyne, 2016	WSP, WCP	Arafura Sea, east of Wessel Islands, Northern Territory, Australia.	Fyler et al. (2009), Caira and Jensen (2017)	**1**¶
*A. odonoghuei* Campbell & Beveridge, 2002	SAM AHC 22699	SAM AHC 22699	*Urolophus expansus*	EIO	Holdfast Bay, South Australia	Campbell and Beveridge (2002)	1§
A. odonoghuei*†	NR	NR	*Urolophus lobatus* McKay, 1966	EIO	Esperance, Western Australia	Campbell and Beveridge (2002)	–
*A. olseni* Dailey & Mudry, 1968	USNPC 71216	NR	*Pseudobatos productus* (Ayres, 1854)	ENP, ECP	Newport Beach, California, USA	Dailey and Mudry (1968)	2‡
A. olseni*†	NR	NR	*Pseudobatos planiceps* (Garman, 1880)	ECP, ESP	Lima, Chorrillos, Peru	Iannacone et al. (2011)	–
A. olseni*†	NR	NR	*Urobatis halleri* (Cooper, 1863)	ENP, ECP	Anaheim Bay, California, USA	Appy and Dailey (1973)	–
A. olseni*†	NR	NR	*Urobatis halleri*	ENP, ECP	Puerto Peñasco, Sonora, Mexico	Friggens and Brown (2005)	–
*A. omanense* Maleki, Malek & Palm, 2019	ZCUOK 117	ZCUOK 118–122; ZUTC Platy. 1330–1334, 1 SEM voucher ZUTC Platy. 1335	Gymnura cf. poecilura**	NIO, EIO, WCP, WNP	Chabahar coast, Gulf of Oman, Iran	Maleki et al. (2019)	**1**¶
A. omanense*	NR	NR	Gymnura cf. poecilura	NIO, EIO, WCP, WNP	Bandar Abbas, Persian Gulf, Iran	Maleki et al. (2019)	–
*A. parviuncinatum* Young, 1954	USNPC 49095	NR	*Urobatis halleri*	ENP, ECP	San Diego Bays, California, USA	Young (1954)	8‡
A. parviuncinatum*	NR	NR	*Gymnura marmorata* (Cooper, 1864)	ECP	San Diego Bays, California, USA	Young (1954)	–
*A. parviuncinatum*†	NR	NR	*Urobatis halleri*	ENP, ECP	Puerto Peñasco, Sonora, Mexico	Friggens and Brown (2005)	–
*A. parvum* Manger, 1972	NR	NR	*Dipturus batis*	ENA	Faxa Bay, Western coasts Iceland	Manger (1972)	6§
*A. paulum* Linton, 1890	NR	USNPC 07683, 35882, 71351, 71352.	*Bathytoshia centroura*	WSA, WCA, WNA	Woods Hole, Massachusetts, USA	Linton (1890), Vardo-Zalik and Campbell (2011)	1(8,9,5)‡
A. paulum*†	NR	NR	*Raja eglanteria*	WCA, WNA	Chesapeake Bay, Virginia, USA	Campbell (1969)	–
A. paulum*†	NR	NR	*Hypanus americanus*	WSA, WCA, WNA	Chesapeake Bay, Virginia, USA	Campbell (1969)	–
*A. pearsoni* Williams, 1962	NR	NR	*Orectolobus maculatus* (Bonnaterre, 1788)	EIO, WSP	Hastings Point NSW, Australia	Williams (1962),Campbell and Beveridge (2002)	1§
*A. persicum* Maleki, Malek & Palm, 2019	ZCUOK 135	ZCUOK 136–137; ZUTC Platy. 1351–1352, 1 SEM voucher ZCUOK 142, 1 SEM voucher ZUTC Platy. 1353	Gymnura cf. poecilura**	NIO, EIO, WCP, WNP	Bandar Abbas, Persian Gulf, Iran	Maleki et al. (2019)	**1**¶
*A. peruviense* Reyda, 2008	USNPC 99945	USNPC 99946; LRP 4108–4111 (including whole mounts and SEM specimens); MZUSP 6393a–6393b; MHNP 2335	*Potamotrygon motoro* (Müller & Henle, 1841)	WSA, WCA	Madre de Dios River at Boca Manu, Madre de Dios Department, Peru	Reyda (2008)	**1(8)**¶
*A. pichelinae* Campbell & Beveridge, 2002	SAM AHC 28229	SAM AHC 28230	*Myliobatis tenuicaudatus*	EIO, WSP	Devonport, Tasmania	Campbell and Beveridge (2002)	4§
*A. pichelinae*†	NR	NR	*Myliobatis tenuicaudatus*	EIO, WSP	Bunbury, Western Australia	Campbell and Beveridge (2002)	–
*A. pintanensis* Wang, 1984	NR	NR	Neotrygon kuhlii**	WSP	Fujian Province, China	Wang (1984)	**4**¶
*A. polytesticularis* Wang & Yang, 2001	PRLXU	NR	*Squalus* sp.**	?	Xiamen, Fujiari, China	Wang and Yang (2001)	4§
*A. ponticum* Léon-Borcea, 1934	NR	NR	*Raja clavata*	ENA, MED, ECA, ESA, WIO	Agigéa, Black Sea	Léon-Borcéa (1934)	?¶
A. ponticum*	NR	NR	*Dasyatis pastinaca*	ENA, MED, ECA	Agigéa, Black Sea	Léon-Borcéa (1935)	–
*A. popi* Fyler, Caira & Jensen, 2009	QM G231350	QM G231351–G231352; USNPC 101959–101960; LRP 4323–4324; cross sections QM G231353; SEM LRP 4329–4330, 4325–4326, hologenophores LRP 4331, 4332	*Urogymnus acanthobothrium*	WSP, WCP	Arafura Sea, east of Wessel Islands, Northern Territory, Australia.	Fyler et al. (2009), Caira and Jensen (2017)	**2**¶
*A. ppdeleoni* Zaragoza-Tapia, Pulido-Flores & Monks, 2020	CNHE 11253	CNHE 11254; HWML 216260	*Hypanus dipterurus*	ECP	Bahía de Chamela, Jalisco, Mexico	Zaragoza-Tapia et al. (2020)	**2**¶
*A. psammobati* Carvajal & Goldstein, 1969	USNPC 71357	USNPC 71358	*Psammobatis scobina* (Philippi, 1857)	ESP	South Pacific Ocean, between Papudo and Talcahuano, Chile	Carvajal-G. and Goldstein (1969)	5‡
A. psammobati*†	NR	CH-MHNJP 342a, 342b	*Sympterygia brevicaudata*	ECP, ESP	Callao, Lima, Peru	Tantaleán-Vidaurre (1991)	–
*A. puertecitense* Caira & Zahner, 2001	CNHE 4175	CNHE 4176; USNPC 90843; LRP 2105–2106	*Heterodontus francisci*	ECP, ESP	Puertecitos, Gulf of California, Mexico	Caira and Zahner (2001)	**4**¶
*A. puntarenasense* Marques, Brooks & Monks, 1995	MNHG 20005	MNHG 20006–20007; HWML 38543, CNHE 4176.	*Hypanus longus*	ECP	Punta Morales, Puntarenas Province, Costa Rica	Marques et al. (1995)	2‡
*A. quadripartitum* Williams, 1968	NR	NR	*Leucoraja naevus* (Müller & Henle, 1841)	ENA, MED, ECA	North Sea, off Aberdeen	Williams (1968)	2§
*A. quinonesi* Mayes, Brooks & Thorson, 1978	USNPC 74804	USNPC 74805; HWML 74806	*Potamotrygon magdalenae* (Duméril, 1865)	WCA	Magdalena River, Cienaga Jobo, vicinity of San Cristobal, Bolivar, Colombia	Mayes et al. (1978)	5‡
A. quinonesi*†	NR	NR	*Potamotrygon yepezi* Castex & Castello, 1970	WCA	Lake Maracaibo area near El Congo and Represa de Tule, Rio Cachiri, Zulia, Venezuela	Brooks et al. (1981)	–
*A. rajaebatis* (Rudolphi, 1810) Euzet, 1959	NR	NR	Dipturus batis**	ENA	Mediterranean Sea	Rudolphi (1810)	5§
A. rajaebatis*†	NR	NR	*Dipturus oxyrinchus* (Linnaeus, 1758)	ENA, MED, ECA	Sète, France	Euzet (1959)	–
A. rajaebatis*†	NR	NR	*Rostroraja alba* (Lacepède, 1803)	ENA, MED, ECA, ESA, WIO	Sète, France	Euzet (1959)	–
A. rajaebatis*†	NR	NR	*Rostroraja alba*	ENA, MED, ECA, ESA, WIO	Lacépède, France	Euzet (1959)	–
*A. rajaebatis*†	NR	NR	Dipturus batis**	ENA	Sète, France	Euzet (1959)	–
*A. rajaebatis*†	NR	NR	Dipturus batis**	ENA	Roscoff, France	Euzet (1959)	–
*A. rajivi* Ghoshroy & Caira, 2001	CNHE 4038	CNHE 4039; HWML 15552; LRP 2055–2056; USNPC 90461	*Hypanus dipterurus*	ECP	Puertecitos, Gulf of California, Mexico	Ghoshroy and Caira (2001)	2‡
*A. ramiroi* Ivanov, 2005	MACN-Pa 412/1-4	USNPC 92521	*Potamotrygon motoro*	WSA, WCA	Río Colastiné, Santa Fé, Argentina	Ivanov (2005)	**4**¶
*A. ramiroi*†	NR	NR	*Potamotrygon motoro*	WSA, WCA	Río Coronda, Santa Fé, Argentina	Ivanov (2005)	–
*A. regoi* Brooks, Mayes & Thorson, 1981	USNPC 75709	USNPC 75710; HWML 21012, 21013	*Potamotrygon hystrix* (Müller & Henle, 1841)	WSA	Orinoco River Delta, Orinoco River near Los Castillos, Venezuela	Brooks et al. (1981)	5‡
A. regoi*†	NR	NR	*Potamotrygon falkneri* Castex & Maciel, 1963	WSA	Paraná River, Brazil	Lacerda et al. (2008)	–
A. regoi*†	NR	NR	*Potamotrygon motoro*	WSA, WCA	Paraná River, Brazil	Lacerda et al. (2008)	–
*A. rhinobati* Alexander, 1953	USNPC 47858	USNPC 47859	*Pseudobatos productus*	ENP, ECP	Santa Monica Harbor, California, USA	Alexander (1953)	9(5)‡
*A. rhinobati*†	NR	NR	*Pseudobatos productus*	ENP, ECP	Ocean Park Pier, California, USA	Alexander (1953)	–
*A. robertsoni* Campbell & Beveridge, 2002	SAM AHC 28197	SAM AHC 22590, 22591, 22592, 22667, 22714	*Trygonorrhina fasciata*	WSP	Middleton, South Australia	Campbell and Beveridge (2002)	3§
A. robertsoni*†	NR	SAM AHC 28257	*Pristiophorus cirratus*	EIO, WSP	Port Stanvac, South Australia	Campbell and Beveridge (2002)	–
A. robertsoni*†	NR	NR	*Aptychotrema vincentiana*	EIO	North Haven, South Australia	Campbell and Beveridge (2002)	–
A. robertsoni*†	NR	NR	*Aptychotrema vincentiana*	EIO	Goolwa, South Australia	Campbell and Beveridge (2002)	–
A. robertsoni*†	NR	NR	*Dentiraja cerva* (Whitley, 1939)	EIO, WSP	Port Stanvac, South Australia	Campbell and Beveridge (2002)	–
A. robertsoni*†	NR	NR	*Dentiraja cerva*	EIO, WSP	Holdfast Bay, South Australia	Campbell and Beveridge (2002)	–
A. robertsoni*†	NR	SAM AHC 28260	*Urolophus bucculentus* Macleay, 1884	EIO, WSP	Rapid Head, South Australia	Campbell and Beveridge (2002)	–
A. robertsoni*†	NR	SAM AHC 22699	*Urolophus expansus*	EIO	Holdfast Bay, South Australia	Campbell and Beveridge (2002)	–
A. robertsoni*†	NR	SAM AHC 28256	*Urolophus lobatus*	EIO	Esperance, Western Australia	Campbell and Beveridge (2002)	–
*A. robertsoni*†	NR	NR	*Trygonorrhina fasciata*	WSP	Outer Harbour, South Australia	Campbell and Beveridge (2002)	–
*A. robertsoni*†	NR	NR	*Trygonorrhina fasciata*	WSP	North Haven, South Australia	Campbell and Beveridge (2002)	–
*A. robertsoni*†	NR	NR	*Trygonorrhina fasciata*	WSP	Port Stanvac, South Australia	Campbell and Beveridge (2002)	–
*A. robertsoni*†	NR	NR	*Trygonorrhina fasciata*	WSP	Goolwa, South Australia	Campbell and Beveridge (2002)	–
*A. robertsoni*†	NR	NR	*Trygonorrhina fasciata*	WSP	Port Vincent, South Australia	Campbell and Beveridge (2002)	–
*A. robertsoni*†	NR	NR	*Trygonorrhina fasciata*	WSP	Queenscliff, Victoria, Australia	Campbell and Beveridge (2002)	–
*A. robustum* Alexander, 1953	USNPC 47856	USNPC 47857	*Pseudobatos productus*	ENP, ECP	Long Beach Harbor, California, USA	Alexander (1953)	4‡
A. robustum*†	NR	NR	*Pseudobatos planiceps*	ECP, ESP	Trujillo, Peru	Escalante-A. (1986)	–
*A. rodmani* Fyler, Caira & Jensen, 2009	QM G231354	QM G231355–G231357; USNPC 101961–101963; LRP 4333–4335; cross sections QM G231359 G231358); cross sections LRP 4564–4569, 4563), longitudinal sections 4560–4562, 4559, SEM LRP 4336–4339, hologenophores LRP 4340, 4341	*Urogymnus acanthobothrium*	WSP, WCP	Arafura Sea, east of Wessel Islands, Northern Territory, Australia.	Fyler et al. (2009), Caira and Jensen (2017)	**6**¶
*A. rohdei* Campbell & Beveridge, 2002	SAM AHC 28233	SAM AHC 28234	*Urolophus lobatus*	EIO	Esperance, Western Australia	Campbell and Beveridge (2002)	1§
*A. romanowi* Fyler, Caira & Jensen, 2009	QM G231360	QM G231361–231363; USNPC 101964–101966; LRP 4342–4344; cross sections QM G231365, G231364); cross sections LRP 4351–4356, SEM LRP 4345–4348, hologenophores LRP 4350, 4349.	*Urogymnus acanthobothrium*	WSP, WCP	Arafura Sea, east of Wessel Islands, Northern Territory, Australia.	Fyler et al. (2009), Caira and Jensen (2017)	**1**¶
*A. rotundum* Subhapradha, 1955	NR	NR	*Rhynchobatus djiddensis*	WIO, NIO	Madras Coast, India	Subhapradha (1955)	**4**¶
*A. royi* Caira & Burge, 2001	CNHE 4173	CNHE 4174; LRP 2104; USNPC 90842	*Diplobatis ommata*	ECP	Punta Arena, Gulf of California, Mexico	Caira and Burge (2001)	**1**¶
*A. royi*†	NR	NR	*Diplobatis ommata*	ECP	Loreto, Gulfo of California, Mexico	Caira and Burge (2001)	–
*A. rubrum* Bilqees, 1980	NR	SPUK 2002 (syntype)	*Mustelus manazo*	NIO, WCP, WNP	Karachi Coast, Pakistan	Bilqees (1980)	6§
*A. saliki* Fyler & Caira, 2006	MZUM (P) 149	USNPC 96418–96419; LRP 3836-3843 (including cross sections and SEM specimens); MZUM (P) 150; IPMB 77.14.07	*Urogymnus polylepis*	NIO, WCP	Off Kampung Abai, Kinabatangan River, Sabah, Malaysia	Fyler and Caira (2006)	1§
*A. santarosaliense* Caira & Zahner, 2001	CNHE 4177	CNHE 4178; USNPC 90844; LRP 2107	*Heterodontus mexicanus* Taylor & Castro-Aguirre, 1972	ECP, ESP	Santa Rosalia, Gulf of California, Mexico	Caira and Zahner (2001)	**3**¶
*A. satyanarayanaraoi* Sanaka, Vijaya Lakshmi & Hanumantha Rao, 1993	DZAUW	NR	*Glaucostegus granulatus* (Cuvier, 1829)	NIO	Waltair coast, India	Sanaka et al. (1993)	4§
*A. schalli* Vardo-Zalik & Campbell, 2011	USNPC 103820	USNPC 103821–103826	*Mustelus canis*	WNA, WCA, WSA	Gulf of Mexico	Vardo-Zalik and Campbell (2011)	**1**¶
A. schalli*	NR	NR	*Mustelus norrisi* Springer, 1939	WNA, WCA, WSA	Gulf of Mexico	Vardo-Zalik and Campbell (2011)	–
*A. semnovesiculum* Verma, 1928	ZIMC	NR	*Pastinachus sephen*	NIO	Allahabad (Ganges and Jumna), India	Verma (1928)	2§
*A. semnovesiculum*†	NR	NR	Pastinachus sephen**	NIO	Fog Bay, Timor Sea, North Australia	Campbell and Beveridge (2002)	–
*A. semnovesiculum*†	NR	NR	Pastinachus sephen**	NIO	Nickol Bay, Western Australia	Campbell and Beveridge (2002)	–
*A. septentrionale* Baer & Euzet, 1962	NR	NR	*Dipturus batis*	ENA	Atlantic, Nort Sea	Baer and Euzet (1962), Baer (1948), Euzet (1959)	3§
A. septentrionale*	NR	NR	*Dipturus oxyrinchus*	ENA, MED, ECA	Atlantic, Nort Sea	Williams (1969)	–
*A. sinaloaensis* Zaragoza-Tapia, Pulido-Flores & Monks, 2020	CNHE 11257	CNHE 11258; HWML 216262	*Hypanus longus*	ECP	La Puntilla, Mazatlán, Sinaloa, Mexico	Zaragoza-Tapia et al. (2020)	**2**¶
*A. soberoni* Ghoshroy & Caira, 2001	CNHE 4040	CNHE 4041–4042; HWML 15548; LRP 2057–2059; USNPC 90462	*Hypanus dipterurus*	ECP	Puertecitos, Gulf of California, Mexico	Ghoshroy and Caira (2001)	6‡
*A. soberoni*†	NR	NR	*Hypanus dipterurus*	ECP	Bahía de Los Angeles, Gulf of California, Mexico	Ghoshroy and Caira (2001)	–
*A. soniae* Zaragoza-Tapia, Pulido-Flores, Violante-Gonzalez & Monks, 2019	CNHE 11136	CNHE 11137; HWML 139978; CHE P00081	*Narcine entemedor*	ECP	Bahía de Acapulco, Playa Las Hamacas, Guerrero, Mexico	Zaragoza-Tapia et al. (2019)	**2**¶
*A. southwelli* Subhapradha, 1955	NR	NR	Rhinobatos schlegelii** Müller & Henle, 1841	WNP	Madras Coast, India	Subhapradha (1955)	1§
*A. sphaera* Maleki, Malek & Palm, 2013	ZUTC 1298	ZUTC 1299–1307), SEM vouchers ZUTC 1308–1309; IPCAS C–641; ZMB E7560	Pastinachus cf. sephen**	NIO	Persian Gulf, Iran	Maleki et al. (2013)	**2**¶
*A. stefaniae* Franzese & Ivanov, 2018	MACN-Pa 624	MACN-Pa 625/1–6, 626/1–3, 627/1, 628/1–2; IPCAS C-786; LRP 9403–9410	*Discopyge tschudii* Heckel, 1846	ESP, WSA	Coastal waters off Mar Chiquita City, Buenos Aires Province	Franzese and Ivanov (2018)	**1**¶
*A. stefaniae*†	NR	NR	*Discopyge tschudii*	ESP, WSA	Coastal waters off Villa Gesell, Argentina	Franzese and Ivanov (2018)	–
*A. stefaniae*†	NR	NR	*Discopyge tschudii*	ESP, WSA	Off San Clemente del Tuyú, Argentina	Franzese and Ivanov (2018)	–
*A. stefaniae*†	NR	NR	*Discopyge tschudii*	ESP, WSA	Off Camarones, Argentina	Franzese and Ivanov (2018)	–
*A. stevensi* Campbell & Beveridge, 2002	SAM AHC 28198	SAM AHC 28199	*Trygonorrhina fasciata*	WSP	Marion Bay, South Australia	Campbell and Beveridge (2002)	2§
*A. stevensi*†	NR	NR	*Trygonorrhina fasciata*	WSP	Goolwa, South Australia	Campbell and Beveridge (2002)	–
*A. stevensi*†	NR	NR	*Trygonorrhina fasciata*	WSP	Coorong, Australia	Campbell and Beveridge (2002)	–
*A. tasajerasi* Brooks, 1977	USNPC 73961	USNPC 73962; HWML 20261	*Styracura schmardae*	WCA	Caribbean Sea, La Cienaga, Magdalena, Colombia	Brooks (1977)	2‡
A. tasajerasi*†	NR	NR	*Hypanus guttatus* (Bloch & Schneider, 1801)	WSA, WCA	Lake Maracaibo, Venezuela	Mayes and Brooks (1981)	–
*A. terezae* Rego & Dias, 1976	CHIOC 31.215c	CHIO 10.847, 10.994, 31.412a-b, 31.215a-b	*Potamotrygon motoro*	WSA, WCA	Rio Salobra, Mato Grosso, Brazil	Rêgo and Luna Dias (1976)	4‡
*A. tetabuanense* Reyda & Caira, 2006	MZUM (P) 184(h)	USNPC 97470–97471; LRP 3869–3873 (including cross sections and SEM specimens); MZUM (P) 185(p)–186(p); IPMB 77.08.18	*Pateobatis uarnacoides*	NIO, WCP	Off Kampung Tetabuan, Sabah, Malaysia	Reyda and Caira (2006)	**2**¶
*A. thomasae* Campbell & Beveridge, 2002	SAM AHC 28201	SAM AHC 22676	*Aptychotrema vincentiana*	EIO	Musgrave Shoal, South Australia	Campbell and Beveridge (2002)	2§
*A. thomasae*†	NR	NR	*Aptychotrema vincentiana*	EIO	Cowell, Australia	Campbell and Beveridge (2002)	–
*A. tortum* (Linton, 1916) Baer & Euzet, 1962	NR	NR	*Aetobatus narinari*	WSA, WCA, WNA, ECA	Woods Hole, Massachusetts, USA	Linton (1916)	3‡
*A. tortum*†	NR	NR	*Aetobatus narinari*	WSA, WCA, WNA, ECA	Caimare Chico, Gulf of Venezuela	Mayes and Brooks (1981)	–
*A. tortum*†	NR	USNPC 70494	*Aetobatus narinari*	WSA, WCA, WNA, ECA	Cape Haze Marine Laboratory, Sarasota, Florida.	Campbell (1970)	–
*A. triacis* Yamaguti, 1952	NR	NR	*Triakis scyllium* Müller & Henle, 1839	WNP	Hamazima, Mie, Japan	Yamaguti (1952)	**4**¶
*A. tripartitum* Williams, 1969	NR	NR	*Raja microocellata* Montagu, 1818	ENA, ECA	English Channel, Plymouth	Williams (1969)	2§
*A. ulmeri* Vardo-Zalik & Campbell, 2011	USNPC 103830	USNPC 103831–103837, 103839, 103842, 103846	*Raja texana*	WCA	Gulf of Mexico	Vardo-Zalik and Campbell (2011)	**1**¶
*A. unilateralis* Alexander, 1953	USNPC 47855	NR	*Myliobatis californicus*	ENP, ECP	Long Beach Harbor, California, USA	Alexander (1953)	7(2)‡
*A. urogymni* (Hornell, 1912) Southwell, 1925	NR	NR	*Urogymnus asperrimus* (Bloch & Schneider, 1801)	ECA, WIO, NIO, EIO, WSP, WCP, WNP	Gulf of Mannar, India	Hornell (1912), Southwell (1925)	?¶
*A. urolophi* Schmidt, 1973	USNPC 72284	USNPC 72284	*Trygonoptera testacea* Müller & Henle, 1841	WSP	Glenelg Beach near Adelaide, South Australia	Schmidt (1973)	1§
A. urolophi*†	NR	NR	*Urolophus paucimaculatus*	EIO, WSP	Devonport, Tasmania	Campbell and Beveridge (2002)	–
*A. urotrygoni* Brooks & Mayes, 1980	USNPC 75162	USNPC 75163; HWML 20917	*Urobatis venezuelae* Schultz, 1949	WCA	Cartagena, Colombia	Brooks and Mayes (1980)	2‡
A. urotrygoni*†	NR	NR	*Hypanus guttatus*	WSA, WCA	Lake Maracaibo, Venezuela	Mayes and Brooks (1981)	–
A. urotrygoni*†	NR	NR	*Hypanus guttatus*	WSA, WCA	Isla Margarita, Venezuela	Mayes and Brooks (1981)	–
*A. vargasi* Marques, Brooks & Monks, 1995	MNHG 20011	MNHG 20012–20013; HWML 38545	*Hypanus longus*	ECP	Punta Morales, Puntarenas Province, Costa Rica	Marques et al. (1995)	2‡
*A. vidali* Zaragoza-Tapia, Pulido-Flores, Violante-Gonzalez & Monks, 2019	CNHE 11134	CNHE 11135; HWML 139979- 139981; CHE P00082	*Narcine entemedor*	ECP	Bahía de Acapulco, Playa Las Hamacas, Guerrero, Mexico	Zaragoza-Tapia et al. (2019)	**6**¶
*A. walkeri* Campbell & Beveridge, 2002	SAM AHC 28219	SAM AHC 28220	Pastinachus sephen**	NIO	Nickol Bay, Western Australia	Campbell and Beveridge (2002)	2§
*A. waltairensis* Uma Maheswari, Sanaka, Vijaya Lakshmi & Hanumantha Rao, 1987	NR	NR	*Himantura uarnak*	WIO, NIO, EIO, WCP	Waltair coast, India	Maheswari et al. (1987)	3§
*A. wedli* Robinson, 1959	NR	DMNZ 194b,c,d, 195–197 (syntype)	*Zearaja nasuta* (Müller & Henle, 1841)	WSP	Petone Beach, New Zealand	Robinson (1959)	4§
*A. wedli*†	NR	NR	*Zearaja nasuta*	WSP	PortobeIlo, Otago Harbour, New Zealand	Robinson (1959)	–
*A. wedli*†	NR	NR	*Zearaja nasuta*	WSP	South Island, off Lyttelton, New Zealand	Campbell and Beveridge (2002)	–
*A. westi* Vardo-Zalik & Campbell, 2011	USNPC 103841	USNPC 103838, 103840, 103843–103845, 103847	*Raja texana*	WCA	Gulf of Mexico	Vardo-Zalik and Campbell (2011)	**1**¶
*A. woodsholei* Baer, 1948	NR	MNHG 40028 (syntype)	*Bathytoshia centroura*	WSA, WCA, WNA	Woods Hole, Massachusetts, USA	Baer (1948), Vardo-Zalik and Campbell (2011)	2(7)‡
*A. woodsholei*†	NR	NR	*Bathytoshia centroura*	WSA, WCA, WNA	Western North Atlantic	Goldstein (1964)	–
*A. xiamenensis* Yang & Lin, 1994	NR	NR	Rhynchobatus djiddensis**	WIO, NIO	Xiamen, South Fujian, China	Yang (1994)	5§
*A. zainali* Fyler & Caira, 2006	MZUM (P) 151	USNPC 96420–96422; LRP 3844-3849 (including cross sections and SEM specimens); MZUM (P) 152–153; IPMB 77.14.08	*Urogymnus polylepis*	NIO, WCP	Off Kampung Abai, Kinabatangan River, Sabah, Malaysia	Fyler and Caira (2006)	1§
*A. zapterycum* Ostrowski de Nuñez, 1971	MACN-Pa 214/1	NR	*Zapteryx brevirostris* (Müller & Henle, 1841)	WSA	Mar del Plata, Buenos Aires, Argentina	Ostrowski de Núñez (1971)	2‡
*A. zapterycum*†	NR	MACN-Pa 214/1-2, 214/4–5, 629/1, 630/1–3, 631/1–4, 632/1–4; IPCAS C-787; LRP 9411–9417	*Zapteryx brevirostris*	WSA	Coastal waters off Villa Gessel, Argentina	Franzese and Ivanov (2018)	–
*A. zapterycum*†	NR	NR	*Zapteryx brevirostris*	WSA	La Lucila del Mar, Argentina	Franzese and Ivanov (2018)	–
*A. zapterycum*†	NR	NR	*Zapteryx brevirostris*	WSA	Puerto Quequén, Argentina	Franzese and Ivanov (2018)	–
*A. zapterycum*†	NR	NR	*Zapteryx brevirostris*	WSA	Puerto Pirámides, Argentina	Franzese and Ivanov (2018)	–
*A. zimmeri* Fyler, Caira & Jensen, 2009	QM G231366	QM G231367–G231369; USNPC 101967–101969; LRP 4357–5358; cross sections QM G231371, G231370); cross sections LRP 4364–4366, SEM LRP 4359–4361, hologenophores LRP 4363, 4362	*Urogymnus acanthobothrium*	WSP, WCP	Arafura Sea, east of Wessel Islands, Northern Territory, Australia.	Fyler et al. (2009), Caira and Jensen (2017)	**1**¶
*A. zschokkei* Baer, 1948	MHNG 88/39	NR	Torpille (common name)**	?	Naples, Italy	Baer (1948)	6§
A. zschokkei*†	NR	NR	*Torpedo marmorata*	ENA, MED, ECA, ESA	Adriatic Sea, Mediterranean Sea	Goldstein (1967)	–
A. zschokkei*†	NR	NR	*Torpedo torpedo*	ENA, MED, ECA	Sète, France	Euzet (1959)	–
A. zschokkei*†	NR	NR	*Torpedo torpedo*	ENA, MED, ECA	Adriatic Sea, Mediterranean Sea	Goldstein (1967)	–

**Figure 2.**
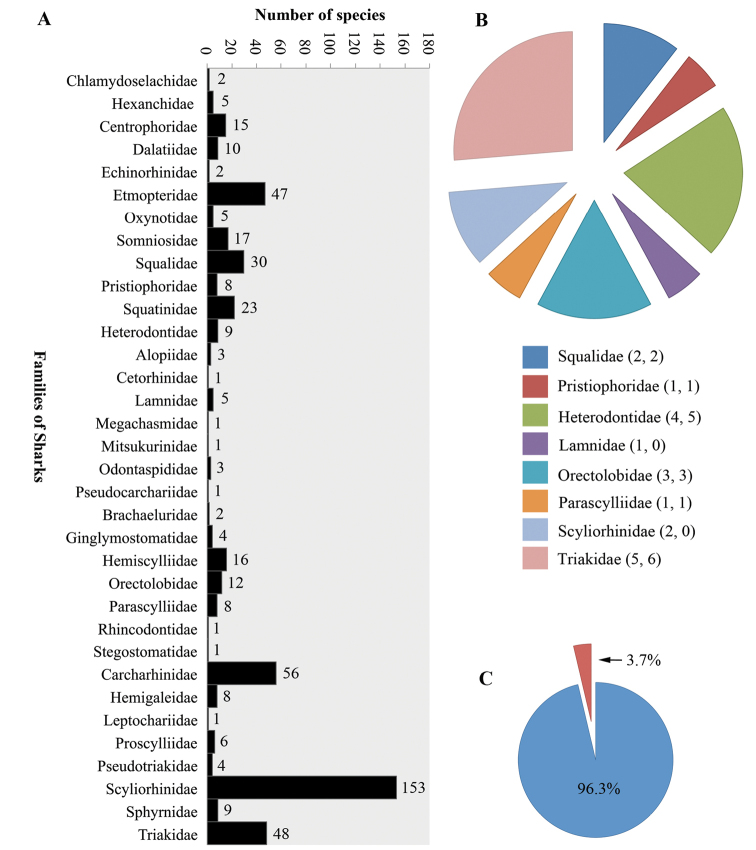
Families of sharks: **A** number of species of sharks per family **B** number of species of sharks parasitized by species of *Acanthobothrium*. Note: The first number within parentheses corresponds to the number of species of shark that have been reported as hosts of *Acanthobothrium* and the second is the number of species that have been described from that Family **C** percentage of species of shark reported to be parasitized within the total number of families of sharks- note: Red color = parasitized; Blue color = not parasitized.

**Figure 3.**
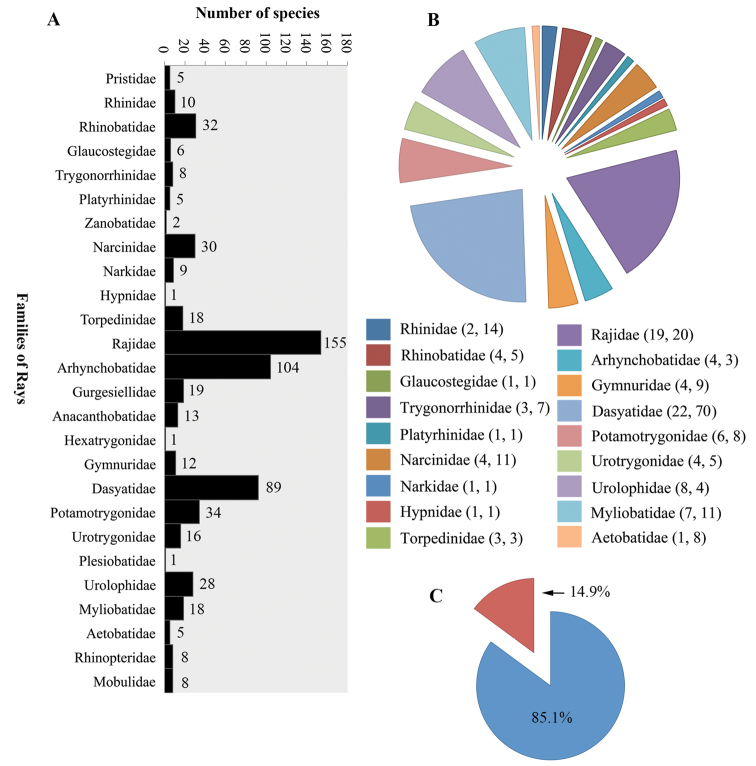
Families of rays: **A** number of species of rays per family **B** number of species of rays parasitized by species of *Acanthobothrium*. Note: The first number within parentheses corresponds to the number of species of ray that have been reported as hosts of *Acanthobothrium* and the second is the number of species that have been described from that Family **C** percentage of species of rays reported to be parasitized within the total number of families of rays- note: Red color = parasitized; Blue color = not parasitized.

The type localities where species of *Acanthobothrium* have been reported is shown in Figure 1. The currently known diversity of sharks comprises 517 species (34 families); of these, 19 species of sharks (eight families) have been reported to be parasitized by species of *Acanthobothrium* (Fig. 2). Eighteen of the 201 valid species have been described from sharks. The families of sharks that have the highest number of reports are Orectolobidae (three different species of *Acanthobothrium*), Heterodontidae (five species) and Triakidae (six species) (Fig. 2B). In contrast, currently known diversity of rays comprises 637 species (26 families); of these, 95 species (18 families) have been reported to be parasitized by species of *Acanthobothrium* (Fig. 3). Of the 201 valid species of *Acanthobothrium*, 182 have been described from rays. The families of rays that have the highest number of reports are Rajidae (20 species of *Acanthobothrium*) and Dasyatidae (70 species) (Fig. 3B).

Species of *Acanthobothrium* are not evenly grouped in the different categories. In Category 1 there are 55 species, 44 in Category 2, 19 in Category 3, 37 in Category 4, 17 in Category 5, 14 in Category 6, four in Category 7, four in Category 8, and three in Category 9. Although there is a Category 10, species in that category also are in grouped with those in Category 8 because their characteristics are thought to fall into both categories (Table 1). The categories of four species of *Acanthobothrium* were classified as unknown (“?”) because the original descriptions do not have sufficient information for assignment in one of the ten categories (Table 1).

## Discussion

Currently, 517 species of sharks have been described worldwide with 3.7% (19 of the 517 species) have been reported as hosts for species of *Acanthobothrium* (Fig. 2C). In contrast, 637 species of rays have been described with 14.9% (95 of the 637 species) have been reported as hosts for species of *Acanthobothrium* (Fig. 3C). Estimates of cestode diversity in elasmobranchs discussed by Caira (2011) assumes that the fauna of cestodes of a species of elasmobranchs does not vary substantially across in its distribution. Knowledge of life cycles are essential in understanding the distribution of species of *Acanthobothrium*; however, for this study it is assumed that the distribution of adults of these parasites normally is limited to that of its normal definitive host. Thus, it is hypothesized that the limits of the distribution of the host limits the species of its parasites to the same biogeographic regions proposed for the distribution of elasmobranchs by Last et al. (2016b). It is recognized that an infected elasmobranch could move outside of the region where it has been designated, but until an extension to its distribution has been reported, it must be assumed that the normal distribution for each species of parasite also is that same designated region. The information in the table will be subject to future research, not forgetting that there is a lack of knowledge of the life cycle of the species of *Acanthobothrium*; a partial life cycle of a single species has been reported (Holland and Wilson 2009). Publication of molecular sequences for more species will provide new discoveries in this subject.

**Figure 4.**
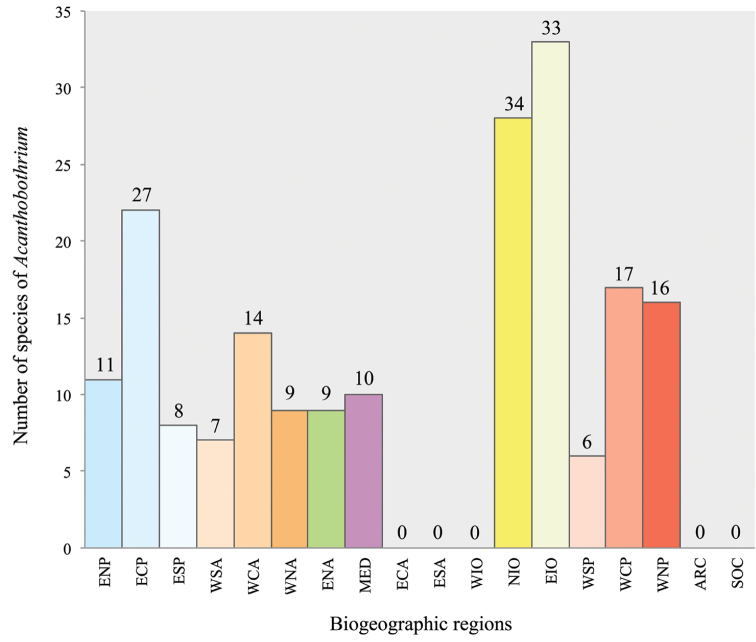
Number of species of *Acanthobothrium* reported from elasmobranchs in each biogeographic region (Last et al. 2016b).

The information in the Figures 1 and 4 indicates that there is an absence of reports from several regions of the world, such as ECA, ESA, WIO, ARC, and SOC. According to the percentages of species of elasmobranchs that have been reported as hosts of species of *Acanthobothrium*, we can infer that there are still many new species of *Acanthobothrium* to be discovered. In the GenBank database records, molecular sequences of only 16 of the 201 species of *Acanthobothrium* have been reported. However, more molecular information about species of *Acanthobothrium* is required for future analyzes, both for identification and life cycle studies; these would provide more solid information for delimiting distributions.

In Table 1, *Acanthobothrium chilensis* Rêgo, Vicente & Herrera, 1968, was included for reference, although it was described from a fish, *Sarda chiliensis* (Cuvier, 1832) (Perciformes: Scombridae) (see Rêgo et al. 1968). Extensive recent studies of this species of fish (Chero et al. 2016; Luque et al. 2016) failed to report *A. chilensis*; there is only the report by Rêgo et al. (1968). The report of the host for this species of *Acanthobothrium* likely is an accidental infection and not a normal host.

According to Fyler et al. (2009) and Franzese and Ivanov (2018), species of *Acanthobothrium* appear to exhibit oioxenous specificity for their elasmobranch hosts. In the present metadata analysis, for species exclusively in elasmobranchs, 83% of the species of *Acanthobothrium* show remarkable host specificity for their definitive host, and thus, should be considered to be an oioxenous species. In contrast, 34 of the 200 species (17%) of *Acanthobothrium* have been reported in more than one species of elasmobranch (Table 1). However, with the metadata analysis of the distribution of the hosts and the reports of the species of *Acanthobothrium*, 45 of the type specimens of *Acanthobothrium* require confirmation of the host (Table 1) because some appear to be problematic identifications and other hosts were reported as “cf.” or only as an unidentified member of a particular genus In addition, there are reports of species of *Acanthobothrium* that suggest misidentification of the parasites; these should reevaluated in future studies. To mention obvious cases, *A. batailloni* has been reported from the Mediterranean Sea and from the Pacific coast of Peru and Chile and *A. brevissime* has been reported from the Gulf of Mexico and the Pacific coast of Peru.

The categorical method developed by Ghoshroy and Caira (2001) was proposed in order to delimit the number of taxonomic comparisons when describing new species. Using the method of Ghoshroy and Caira (2001), which focused only on species from the Americas, Fyler and Caira (2006) later applied the same methodology to biodiversity data for species from other regions; those works are augmented by this study. Of the 201 known species of *Acanthobothrium*, 13 have been classified in more than one category (see category designations in Table 1) because some characteristics of those species overlap with those of more than one category (see descriptions found in Zschokke 1888; Linton 1890; Baer 1948; Alexander 1953; Euzet 1955; Riser 1955; Yamaguti 1959; Goldstein 1964; Williams 1969; Goldstein et al. 1969; Appy and Dailey 1973; Severino and Sarmiento 1979; Marques et al. 1997; Reyda 2008). This does not decrease the usefulness of the categorical method as a tool for the initial stages in identification.

Having more information, such as molecular sequences, could solve some problems in identification, such as the two cases mentioned above. A species of *Acanthobothrium* that has been assigned to more than one category suggests that the categories still need some refining, or it is an example of cryptic species that cannot be distinguished without molecular information. However, molecular information cannot replace morphological descriptions of species. One reason is the lack of material for sequencing of the vast majority of already-known species. Morphology also augments molecular data in studies of the phylogeny of platyhelminths (Zamparo et al. 2001; Littlewood 2008). A complete phylogenetic hypothesis based on total evidence (morphological and molecular data) such as that of Littlewood (2008) for any major group of cestodes is still distant. Until that time, a categorical method provides the easiest and most direct method for selection of a group of species similar to a new species of *Acanthobothrium*. This updated database includes the category designation for each species described to date will be an important tool for the future taxonomic studies.
